# *FOPBIE*: Multi-image cipher based on the random walk of fleet of pawns on the large hypothetical chessboard and chaotic system

**DOI:** 10.1371/journal.pone.0295060

**Published:** 2024-06-13

**Authors:** Muhammad Akram, Shahzad Ali, Jarallah Alqahtani, Nadeem Iqbal, Ali Alqahtani, Atif Ikram

**Affiliations:** 1 Department of Computer Science, College of Computer Science and Information Systems, Najran University, Najran, Saudi Arabia; 2 Department of Computer Science, Jouf University, Sakaka, Kingdom of Saudi Arabia; 3 Department of Computer Science, College of Computer Science and Information Systems, Najran University, Najran, Saudi Arabia; 4 Department of Computer Science & IT, The University of Lahore, Lahore, Pakistan; 5 Department of Networks and Communications Engineering, College of Computer Science and Information Systems, Najran University, Najran, Saudi Arabia; 6 Faculty of Ocean Engineering Technology and Informatics, Universiti Malaysia Terengganu, Kuala Terengganu, Malaysia; 7 Department of Computer Science & Information Technology, Faculty of Information Technology, The University of Lahore, Lahore, Pakistan; State University of New York at Oswego, UNITED STATES

## Abstract

In the last two decades or so, a large number of image ciphers have been written. The majority of these ciphers encrypt only one image at a time. Few image ciphers were written which could encrypt multiple images in one session. The current era needs speedy multiple image ciphers to address its varied needs in different settings. Motivated by this dictation, the current study has ventured to write a multi-image cipher based on the fleet of pawns walking in the large hypothetical chessboard. This walk of pawns on the chessboard has been ingeniously linked with transferring the pixels from the plain image to the scrambled image. The confusion effects have been realized through the XOR operation between the scrambled image and the key image. The plaintext sensitivity has been incorporated by embedding the SHA-384 hash codes of the given large combined plain image. Moreover, the Henon map has been employed to spawn the streams of random numbers. Besides, Blum Blum Shub random number generator has been used to further cement the security of the proposed cipher. We got a computational time of 0.2278 seconds and an encryption throughput of 5.5782 MBit/seconds by using the four images with a size of 256×256. Apart from that, the information entropy gained is 7.9993. Lastly, the cipher has been subjected to an array of validation metrics to demonstrate its aversion to the myriad threats from the cryptanalysis savvy. We contend that the proposed work has great potential for some real-world applications.

## 1 Introduction

Images have become a very important entity in the current era. They are being generated, saved, and transmitted in in all over the world. A variety of images exist in different domains as varied as medicine, transportation, research, different government facilities, business, space science, etc. In some special settings, these images assume a lot of importance like the image of some new missile or image of some dignitary. So, such images must be dealt with an extreme care. Hackers, on the other hand, are very curious to exploit any vulnerability they may spot and resultantly to hack these images.

The encryption of images through some algorithms is the most natural solution to this problem. Historically, the cryptosystems like DES, AES, RSA, and IDEA have been in use to carry out the task of encryption. Unfortunately, these cryptosystems are not applied to the image data [1]. The reason for this is due to the fact that images have totally distinct characteristics as compared to the text. These characteristics include large volume, strong inter-pixel relations, the extreme redundancy. Hence, we require radically different machinery to serve this purpose. Fortunately, chaotic maps and systems have proved very useful to generate the streams of random numbers. These maps enjoy some good properties of ergodicity, mixing, randomness, and arbitrariness. Through the exploitation of these properties, we may find tens of hundreds of image ciphers in the literature which have been written. These maps come in many flavors. There are low-dimensional and higher-dimensional maps. The low-dimensional maps consist of one or two streams [2], whereas maps producing more than two streams fall under the category of higher-dimension. Both of these categories have their own pros and cons. The maps of a former category can be easily implemented in some programming settings; They take less time in the generation of data but the generated data is not too much chaotic *per se*. In contrast to that, the maps of the latter category can’t be easily implemented but they render much chaotic and random data. Further, these maps are very time consuming and in many situations, they can’t meet the urgency of the modern day requirements. So, there is a sort of trade-off between these two rival requirements. In our study, in order to satisfy both the efficiency and the security requirements of the cipher, we have selected the Honon map [3] which produces two streams of random numbers. In order to throw more security stuff, Blum Blum Shub random number generator has also been employed [4].

Many multi-image ciphers have been written in the past [5–8]. In the study [5], the authors have written a multi-image cipher using 3D shuffling scrambling and the Haar wavelet transform. The proposed algorithm divides the cube into a 1D array and permutes the order of this array. Besides, wavelet tranform has been carried out on all the layers of the cube. Moreover, the shuffling procedure (3D) has been employed for dismantling the low-frequency coefficient and to reconstructing the cube with high-frequency parts and scrambling the low-frequency coefficient. Diffusion effects have been thrown by invoking the XOR operation. By employing dynamic DNA coding, image hash, and bit-plane decomposition, authors of [6] wrote a multi-image encryption algorithm. Moreover, an improved 3D map has been employed for the generation of random numbers. These random numbers facilitated in decomposing bit plane of the merged image. Moreover, the bit-plane matrix has been replaced in a numerical fashion. In the final stage, dynamic DNA coding was used with the help of random numbers. The cipher was subjected to a comprehensive set of validation metrics in order to demonstrate its robustness. In a yet another work [7], the asymmetric key scheme was improved using the elliptic curve philosophy. Both the receiver as well as sender decide on an elliptic curve using the DHK sharing technique. Apart from that, Generator was kept secret and the corresponding hash value was generated and made it public. Only the authorized members could get the Generator through the usage of the hash value. This scheme was implemented over multiple color images. Besides, the pixels’ values were used to realize the effects of scrambling instead of the random numbers. The multi-image cipher given in [8] differs from the others due to the incorporation of permutation-diffusion operation which is cross-coupled chaotic map-based and two-layered in character. The algorithm so developed has employed left-right flipping, bitwise XOR diffusion and block-shuffling in the first layer through the set of chaotic maps (cross-coupled). Moreover, in the second layer, other set of up-down flipping, bitwise XOR operations and block-shuffling have been carried out in the another set of chaotic maps (cross-coupled). Authors have a contention that their work is resistant to widely used attacks.

Many image ciphers have been developed in the past with some loopholes in their design principles. So, researchers paid increasing attention to them and identified those loopholes for the other researchers. For instance, the work [9] was broken by [10]. This work [9] was carried out by using information entropy (IEAIE). The defects identified by the cryptanalysts include short orbits of the chaotic map employed and the poor sensitivity mechanism based on the concept of information entropy of the given plain image. Another work [11] was broken by [12]. The cryptanalysis work of [12] identified in [11] that the diffusion processes could be broken down into the structure of permutation-diffusion which occurs after the original permutation process was carried out. Besides, these two permutation processes could be merged into one. Moreover, it was identified that chaotic sequences did not depend on the plaintext and ciphertext, hence the keys remained the same. In this way, the encryption scheme with single-, two- and multi-round of permutation-diffusion processes was cracked. Lastly, the proposals were given in order to address the lacunas and the other shortcomings in the design principle of the scheme [11]. Besides, many image ciphers took too much time for encryption and decryption [6, 13, 14]. Hence, measures need to be taken in order to inject more security stuff into the design philosophies of the new image ciphers. Besides, they must be speedier to cater to the rising demands of the modern era.

After taking inspiration from the above analyses, in this study, we have engineered a novel multiple image cipher by using the instruments of Henon map, Blum Blum Shub random number generator, chess piece pawn and SHA-384 hash codes. This cipher can be characterized through the following features.

- A fleet of chesspiece pawns has been employed to realize the confusion effects in the images. Particulary, this fleet randomly walks on the large hypothetical chessboard. This random walk has been ingeniously linked with the shifting of pixels from the plain image to the scrambled image.
- In order to raise the security effects, Blum Blum Shub random number generator and SHA-384 hash codes have been used in the core of the proposed algorithm.
- The comprehensive security and performance analyses and the computer machine simulation vividly indicate the immunity of the cipher from multiple threats; its robustness from the varied threats and its real world applicability.

The rest of the paper has been set like this. Section 2 discusses the building blocks on which the current study rests. These blocks are the Henon map, Blum Blum Shub random number generator, chess game and its piece pawn. Section 3 sheds light on the way, a stream of random numbers has been generated and the proposed multiple images encryption algorithm. Section 4 gives the simulation and machine experimentation. Security and performance analyses have been tackled based on the diverse validation metrics in Section 5. The paper has been wrapped up in the last and sixth section along with the necessary concluding remarks.

## 2 Home work

In this section, three building blocks of the proposed research work, *i.e*., chaotic systems, Blum Blum Shub pseudo-random number generator, game of chess and its piece/player pawn will be discussed here a little bit.

### 2.1 Theory of chaos and chaotic systems

According to the theory of chaos, a minute change in one of the variables (initial conditions and system parameters) causes to carry out a huge change in the resultant values [15]. By complying with this theory, mathematicians and scientists have rendered a plethora of chaotic maps and systems. These maps fall into the categories of low dimensional [16] and high dimensional maps [17]. The maps having one or two streams of random data fall into the former category whereas the ones having more than two streams of random numbers fall into the latter category as described earlier. Image cryptographers normally employ these maps to carry out the diffusion and scrambling operations upon the pixels of the plain image. In this regard, we have chosen the 2D Henon map developed in 1976 [3].

$$\begin{array}{c} \begin{array}{c} x_{n+1}=1-ax^{2}\left(n\right)+y\left(n\right),\\ y_{n+1}=bx\left(n\right) \end{array} \end{array}$$ (1)

In the above equation, *a* and *b* refer to the system parameters and *n* = 0, 1, 2,…. The above map exhibits the behavior of chaoticity for *a* ∈ (0.54, 2), *b* ∈ (0, 1).

### 2.2 Blum Blum Shub pseudo-random number generator

Three scientists named Lenore Blum, Manuel Blum and Michael Shub developed a pseudorandom number generator [4] in 1986 which was inspired by the one-way function of Michael O. Rabin [18]. Taking the last names of these three scientists, Blum Blum Shub was coined as its name. Its mathematical equation is

$$\begin{array}{c} x_{n+1}=x_{n}^{2}\;\text{mod}\;M \end{array}$$ (2)

Here *n* = 0, 1, 2, 3, …‥ In the above equation, *M* = *p*.*q*, where *p* and *q* are the two large prime numbers. Further, the value of *x*_0_ is chosen in such a way that it should be an integer and assumes a relationship of co-prime to *M*. Besides, one of the interesting features of the Blum Blum Shub generator is the possibility that *i*^*th*^ value of *x* can be calculated through the usage of Euler’s theorem.

$$\begin{array}{c} x_{i}=\left(x_{0}^{2^{i}\;\text{mod}\;\lambda \left(M\right)}\right)\;\text{mod}\;M \end{array}$$ (3)

In the above equation, λ corresponds to the Carmichael function.

### 2.3 Chess and pawn

**Fig 1:**
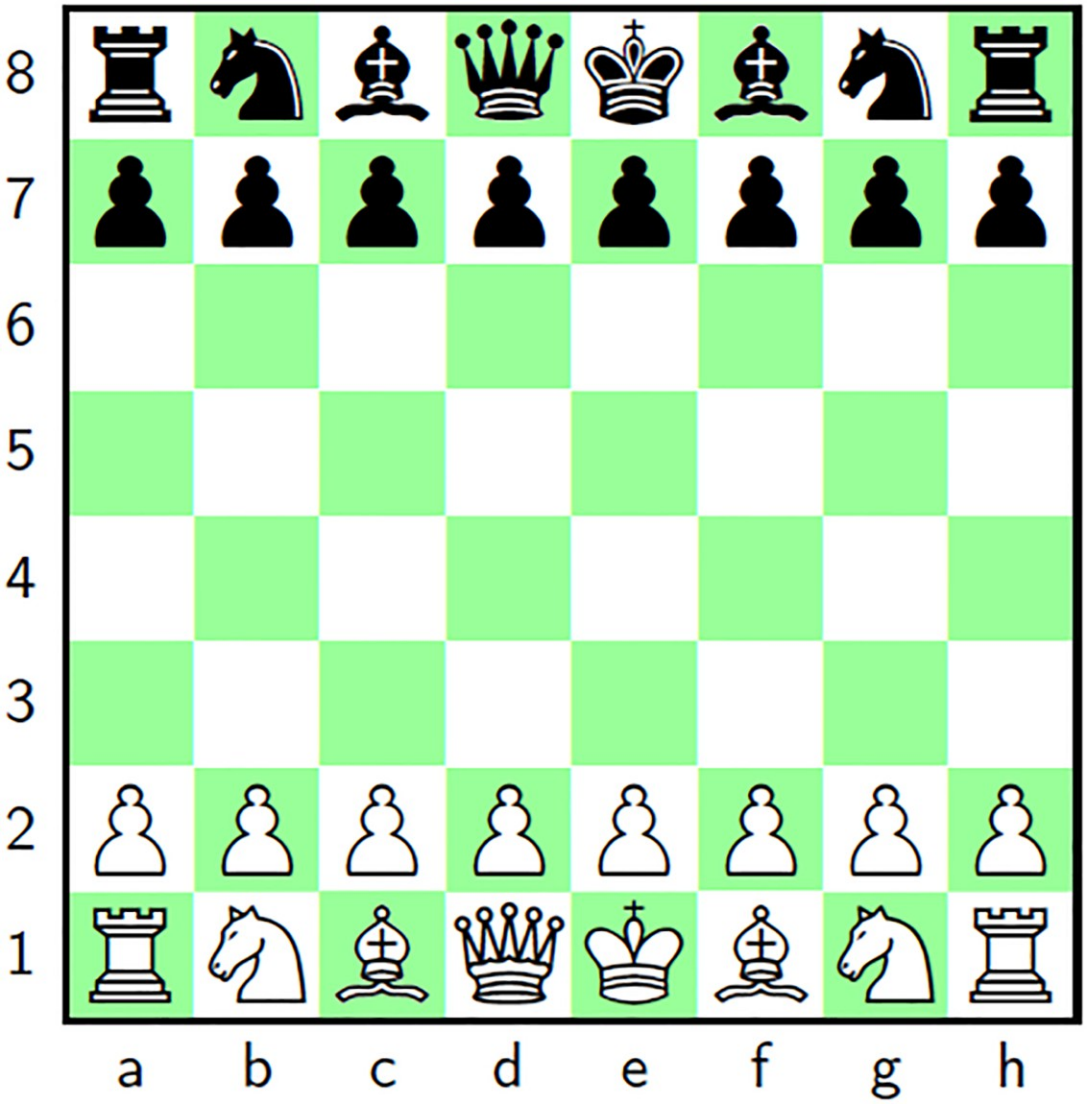
Game of chess: Chess board with pieces.

**Fig 2:**
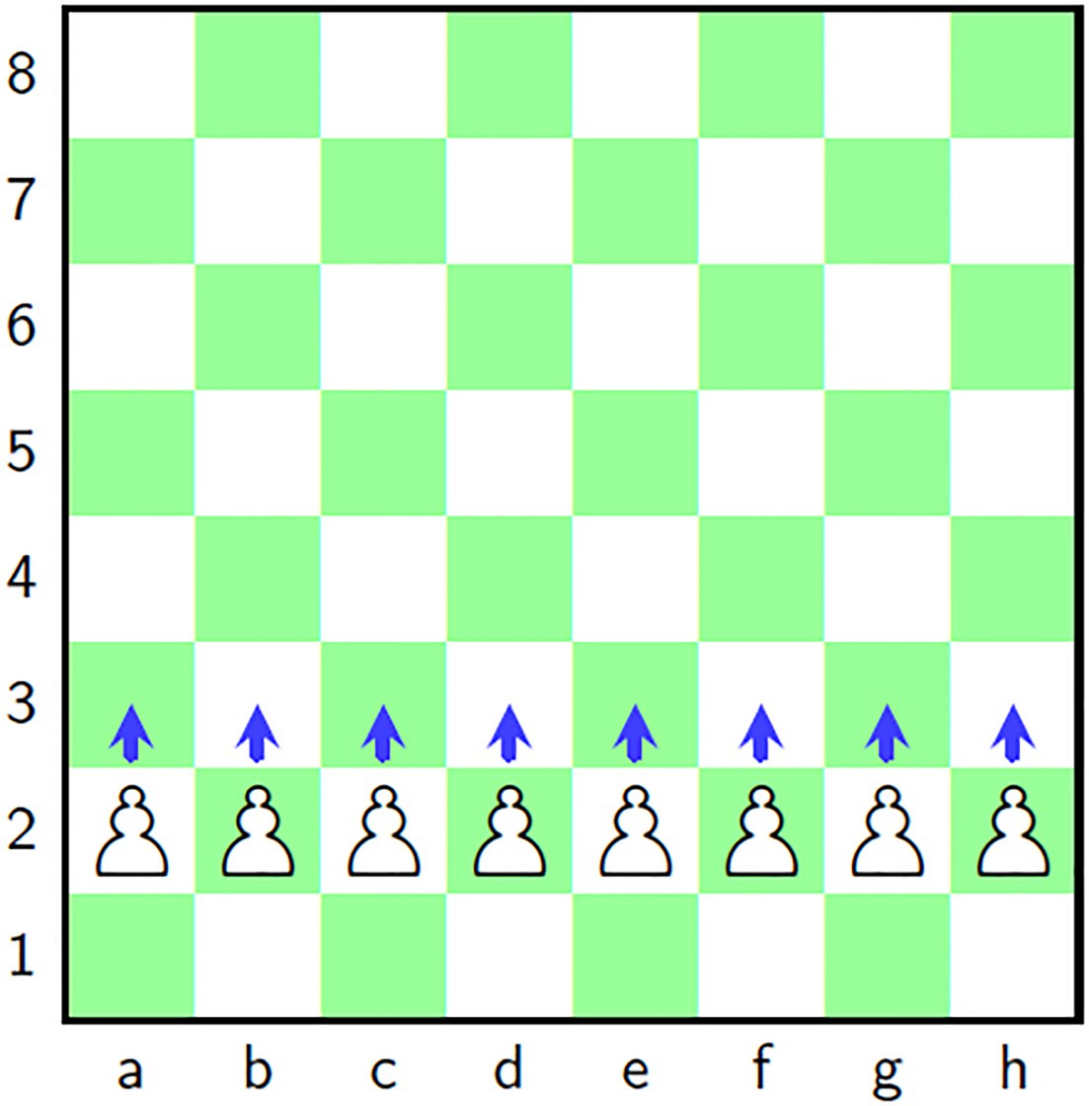
Game of chess: Chess board with moves of Pawn.

Chess [1] is a classical and strategical game in which a board is used, called a chessboard which is an 8 × 8 grid (Fig 1). There are some pieces/players for each of the two players, *i.e*., one king, one queen, two bishops, two knights, two rooks (*aka* castle) and eight pawns. Rows are normally labeled as 1 to 8 and columns *a* to *h*. Fig 1 shows the black colored pieces lying on the rows 7 and 8. Further, the white colored pieces are on the rows 1 and 2. In this game, each piece has a predefined move in some unique way. Besides, each square has a special address. The addresses of the white knights are {1, *b*} and {1, *g*} for instance. Further, the addresses of the white pawns are ${\left\{2,t\right\}}_{t=a}^{h}$. This work has used the chesspiece pawn for the project of scrambling/confusion of the pixels. Pawn can move only one position forward as shown in the Fig 2. To make the chess and Pawn a good fit for the proposed image cipher, a departure has been introduced that size of the chessboard will be dynamically adapted with the size of the input image.

Besides, random numbers will select the particular pawn to move it forward in each iteration of the scrambling project. This movement of the pawns on the chessboard has been associated with the transfer of the pixels from the plain image to the scrambled image.

## 3 Fleet of pawns based image encryption (*FOPBIE*)

**Fig 3:**
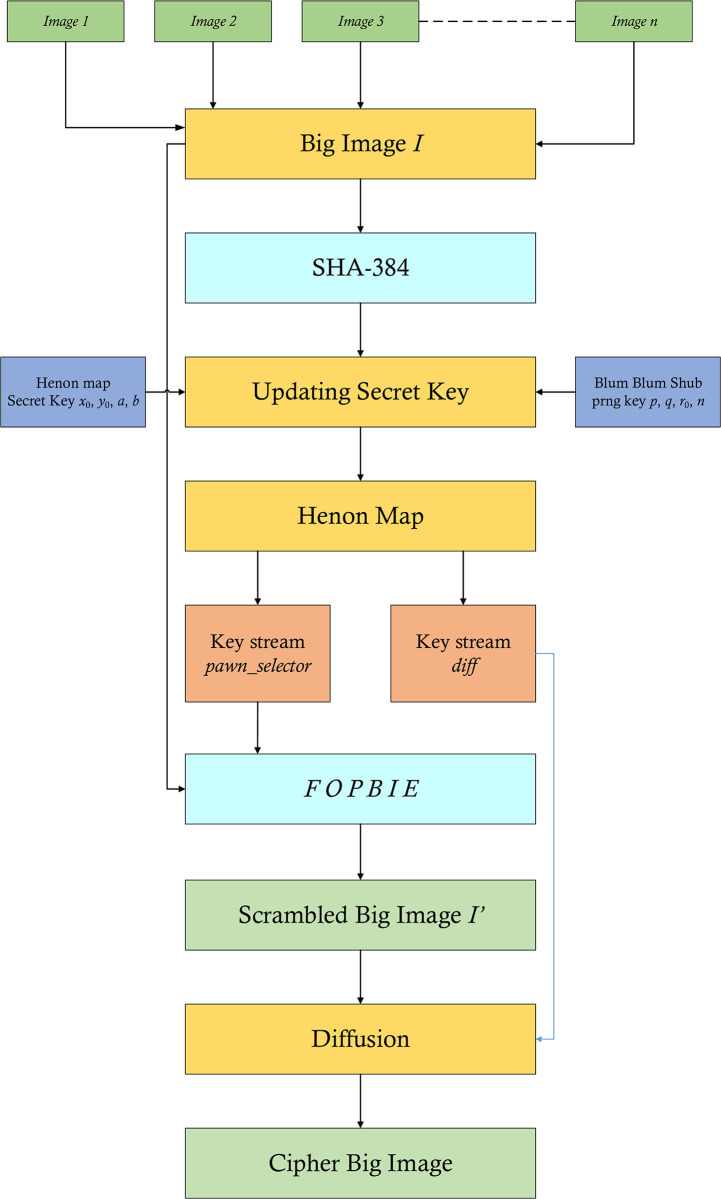
Fleet of pawns based image encryption *FOPBIE*.

The scheme named *FOPBIE* has been designed to address the problem of encryption of multiple images each of the sizes of *m* × *n* in one go. Let the single image formed with the given set of images is *I* and its size is *M* × *N*. The flowchart of this scheme has been drawn in the Fig 3. The suggested cipher for the multiple images consists of some stages. In the first stage, the given images *image 1*, *image 2*, *image 3*,…., *image n* have been joined together to form the Big Image *I*. SHA-384 hash codes have been generated for the Big Image *I*. These codes serve as the fingerprints of the given input images. The different images would spawn the different hash codes which, in turn, would render different streams of random numbers. To put this phenomenon in other words, this serves as the plaintext sensitivity which is a great barrier to the potential threats of differential attacks. The hash codes given by SHA-384 temper the initial values and the system parameters of the Henon map being used. To inject more security stuff, the pseudo-random number generator Blum Blum Shub has been employed. The random numbers given by this generator further temper the initial values of the Henon map. In the second stage, the system parameters and the initial values are given to the Henon map to generate the two streams of random numbers namely *x* and *y*. These two streams have been further translated to the ranges of 1 to *M* and 0 to 255 to comply them with the peculiar logic we have conceived for this project. They have been respectively named as *pawn*_*selector* and *key*_*image*. In the third stage, the stream *pawn*_*selector* has been used to scramble the pixels of the given Big Image *I* and the resultant scrambled image is *I*′ (say). In the fourth and last stage, the scrambled image *I*′ and the stream *key*_*image* have been XORed together to get the final cipher image *Cipher*.

### 3.1 Key stream generation procedure

This subsection illustrates the way, the chaotic data has been spawned. Let *Key* represents the hash codes given by the SHA-384 after it gets applied to the Big Image *I*. We will here use these 8-bit codes *Key*_1_, *Key*_2_, …, *Key*_48_ to temper the initial values and the system parameters of the Henon map. The Algorithm 1 has been called with the parameters $x_{0}^{\prime }$, $y_{0}^{\prime }$, *a*′, *b*′, *Key*, *r*_0_, *p*, *q*, *n*. Here, $x_{0}^{\prime }$, $y_{0}^{\prime }$, *a*′, *b*′ are the initial values and the system parameters for the Henon map. *Key* corresponds to the hash codes given by the Big Image *I*. *r*_0_, *p*, *q* are the initial value and the system parameters of the Blum Blum Shub random number generator and lastly *n* corresponds to the number of random numbers required for the proposed algorithm.

**Algorithm 1**: Calculation of initial values and the system parameters

**Input:** $x_{0}^{\prime }$ , $y_{0}^{\prime }$, *a*′, *b*′, *Key*, *r*_0_, *p*, *q*, *n*

**Output**: *x*_0_, *y*_0_, *a*, *b*

**1** $x_{0}^{\prime \prime }\leftarrow x_{0}^{\prime }+\frac{\sum_{i=1}^{11}\left(Key_{i}⊕Key_{i+1}\right)}{2^{50}},i=1,3,5,\ldots ,11$

**2** $y_{0}^{\prime \prime }\leftarrow y_{0}^{\prime }+\frac{\sum_{i=13}^{23}\left(Key_{i}⊕Key_{i+1}\right)}{2^{50}},i=13,15,17,\ldots ,23$

**3** $a^{\prime \prime }\leftarrow a^{\prime }+\frac{\sum_{i=25}^{35}\left(Key_{i}⊕Key_{i+1}\right)}{2^{50}},i=25,27,29,\ldots ,35$

**4** $b^{\prime \prime }\leftarrow b^{\prime }+\frac{\sum_{i=37}^{47}\left(Key_{i}⊕Key_{i+1}\right)}{2^{50}},i=37,39,41,\ldots ,47$

**5** *M* ← *p*.*q*

**6** **for** *i* ← 0 **to** *n* **do**

**7** $r_{i+1}\leftarrow r_{i}^{2}$ *mod* *M*

**8** $x_{0}\leftarrow x_{0}^{\prime \prime }+\frac{\sum_{i=0}^{n4}\left(r_{i}+r_{i+1}\right)}{2^{50}}$

**9** $y_{0}\leftarrow y_{0}^{\prime \prime }+\frac{\sum_{i=n4+1}^{n2}\left(r_{i}+r_{i+1}\right)}{2^{50}}$

**10** $a\leftarrow a^{\prime \prime }+\frac{\sum_{i=n2+1}^{3n4}\left(r_{i}+r_{i+1}\right)}{2^{50}}$

**11** $b\leftarrow b^{\prime \prime }+\frac{\sum_{i=3n4+1}^{n}\left(r_{i}+r_{i+1}\right)}{2^{50}}$

**Algorithm 2**: Spawning of chaotic vectors

**Input**: *x*_0_, *y*_0_, *a*, *b*, *M*, *N*

**Output**: *pawn*_*selector*, *diff*

**1 for** *index* ← 1 **to** *MN* **do**

**2**  *pawn*_*selector*_*index*_ ← ⌊*mod*(((*abs*(*x*_*index*_) − ⌊*abs*(*x*_*index*_)⌋)) × 10^14^, *M*)⌋ + 1

**3**  *diff*_*index*_ ← ⌊(((*mod*(*abs*(*y*_*index*_) − ⌊*abs*(*y*_*index*_⌋)) × 10^14^, 256)⌋

Here, we explain the Algorithm 1.

Lines (1–4) depict the way, hash codes represented by the *Key* have been used in tempering the initial values ($x_{0}^{\prime },y_{0}^{\prime }$) and the system parameters (*a*′, *b*′) of the map. The set of variables after getting tempered is ($x_{0}^{\prime \prime },y_{0}^{\prime \prime },a^{\prime \prime },b^{\prime \prime }$). Lines (6–7) calculate the stream *r* of *n* random numbers given by the Blum Blum Shub pseudorandom number generator. Lastly, the lines (8–11) further temper the initial values and the system parameters of the Henon map through the usage of this stream *r*. Finally, we get the set (*x*_0_, *y*_0_, *a*, *b*) which will be used to spark the Henon map.

The tempered initial values (*x*_0_, *y*_0_, *a*, *b*) ignite the chaotic map defined in the System (1). Upon finishing the iterations of this map, these sequences ${\left\{x_{t}\right\}}_{t=1}^{MN+\varepsilon }$, ${\left\{y_{t}\right\}}_{t=1}^{MN+\varepsilon }$ have been obtained. Here, (*M*, *N*) is the size of input image. In these two equations, *ϵ* ≥ 500. As the map iterates, its values get matured after passing a certain threshold. Normally, the first *ϵ* values are overlooked.

Invoke Algorithm 2 with the parameters *x*_0_, *y*_0_, *a*, *b*, *M* and *N*. We got the two streams ${\left\{pawn\_selector_{t}\right\}}_{t=1}^{MN}$ and ${\left\{diff_{t}\right\}}_{t=1}^{MN}$ as its output. Moreover, the operator ⌊.⌋ denotes the floor function.

### 3.2 Detailed procedure of image encryption

Call Algorithm 3 with parameters *I*, *pawn*_*selector*. This algorithm is explained as follows.

Find the size of the given image *I* and let it be (*M*, *N*) (Line 1).

Reshape the image *I* to the size of 1×*MN* (Line 2).

Lines (3–5) create the scrambled image *Scrambled* initialized with -1 and its size is *M*×*N*.

**Fig 4:**
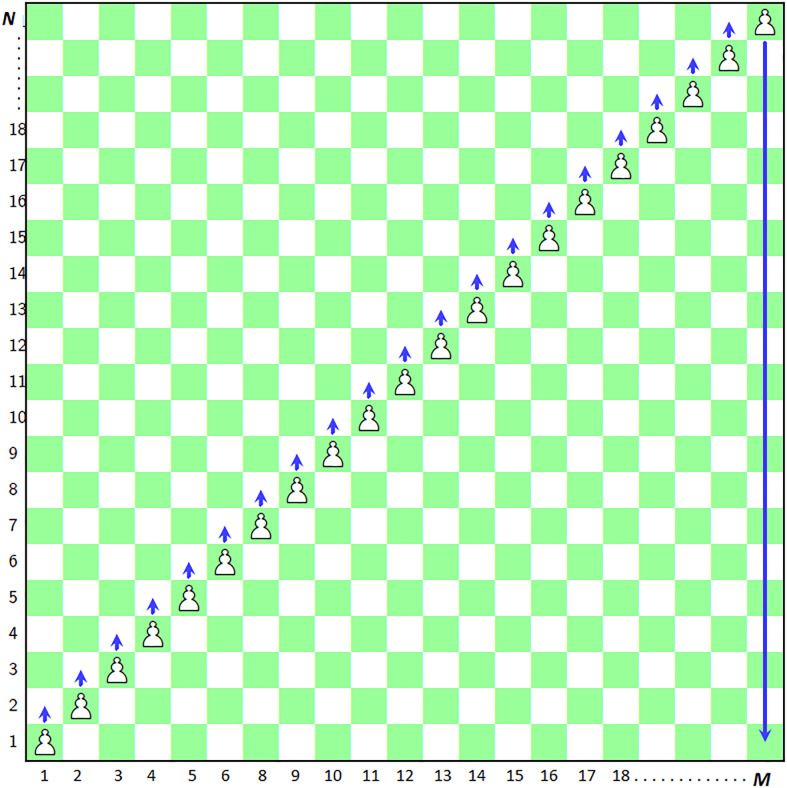
Fleet of pawns based image scrambler.

On the lines (6–7), *ChessBoard* has been initialized as $ChessBoard{\left\{i\right\}}_{i=1}^{N}$ where *i* is the index of the array and *N* corresponds to the maximum height of the board (Fig 4).

#### 3.2.1 Dynamics of scrambling project

Here we will describe the way, the scrambling project has been carried out. To start with, we have four entities at our disposal, *i.e*., input plain image *I*, scrambled image *Scrambled*, 1D array *ChessBoard* and the streams of random numbers *pawn*_*selector*. Only one pawn out of the fleet of pawns moves forward on the chessboard in each iteration. The process of shifting the pixels from the plain image to the scrambled image *Scrambled* has been iterated *MN* times (Lines 8–16). The array *pawn*_*selector* selects the particular pawn which has its turn for movement. Besides, a count has been maintained in the array of *Chessboard*. This array contains the frequency with which each pawn has been moved. As particular pawn moves at some index say *k*. Its corresponding count gets updated by adding 1 to *ChessBoard*(*pawn*_*selector*(*k*)) (Line 16). At the address (*i* = *pawn*_*selector*(*k*), *j* = *ChessBoard*(*pawn*_*selector*(*k*))) of the scrambled image, the *k*^*th*^ pixel from the plain image *I* is shifted (Line 14). This pixel *I*(*k*) has been further marked as -1 (Line 15). Lastly, the lines (17–24) refer to the shifting of the remaining pixels of *I* to the scrambled image *Scrambled* by using the *rpi* (which stands for remaining pixels index). This scrambled image has been finally assigned to the *I*′ (Line 25).

Now reshape the scrambled image *I*′ to 1 × *MN* and diffuse it further by carrying out an XOR operation between it and the stream of random numbers *diff*.

$$\begin{array}{c} Cipher\left(i\right)=I^{\prime }\left(i\right)⊕diff\left(i\right) \end{array}$$ (4)

where 1 ≤ *i* ≤ *MN*. Finally, resize *Cipher* to *M* × *N* to get the final cipher image.

**Algorithm 3**: Scrambling

**Input**: *I*, *pawn*_*selector*

**Output**: *I*′

**1** (*M*, *N*) ←*size*(*I*)

**2** Reshape *I* to the size of 1 × *MN*

**3** **for** *i* ← 1 **to** *M* **do**

**4**  **for** *j* ← 1 **to** *N* **do**

**5**   *Scrambled*(*i*, *j*) ← −1

**6** **for** *i* ← 1 **to** *N* **do**

**7**  *ChessBoard*(*i*) ← *i*

**8** **for** *k* ← 1 **to** *MN* **do**

**9**  *i* ← *pawn*_*selector*(*k*)

**10**  **if** *ChessBoard*(*pawn*_*selector*(*k*) > *N*) **then**

**11**   *ChessBoard*(*pawn*_*selector*(*k*) ← 1

**12**  *j* ← *ChessBoard*(*pawn*_*selector*(*k*))

**13**  **if** *Scrambled*(*i*, *j*) = −1 **then**

**14**   *Scrambled*(*i*, *j*) ← *I*(*k*)

**15**   *I*(*k*) ← −1

**16**  *ChessBoard*(*pawn*_*selector*(*k*)) ← *ChessBoard*(*pawn*_*selector*(*k*)) + 1

**17** *rpi* ← 1

**18** **for** *i* ← 1 **to** *M* **do**

**19**  **for** *j* ← 1 **to** *N* **do**

**20**   **if** *Scrambled*(*i*, *j*) = −1 **then**

**21**    **while** *I*(*rpi* + 1 = −1) **do**

**22**     *rpi* ← *rpi* + 1

**23**    *Scrambled*(*i*, *j*) ← *I*(*rpi* + 1)

**24**   *rpi* ← *rpi* + 1

**25** *I*′ ← *Scrambled*

The decryption algorithm of the encryption is very straightforward. The reason of this stems from the fact that the decryption algorithm is an exact inverse of the steps of the encryption algorithm. So, we will not entertain it here in depth.

### 3.3 Ethics statement

The individual pictured in Fig 8 has provided written informed consent (as outlined in PLOS consent form) to publish their image alongside the manuscript.

## 4 Experimentation through computer simulation

**Fig 5:**
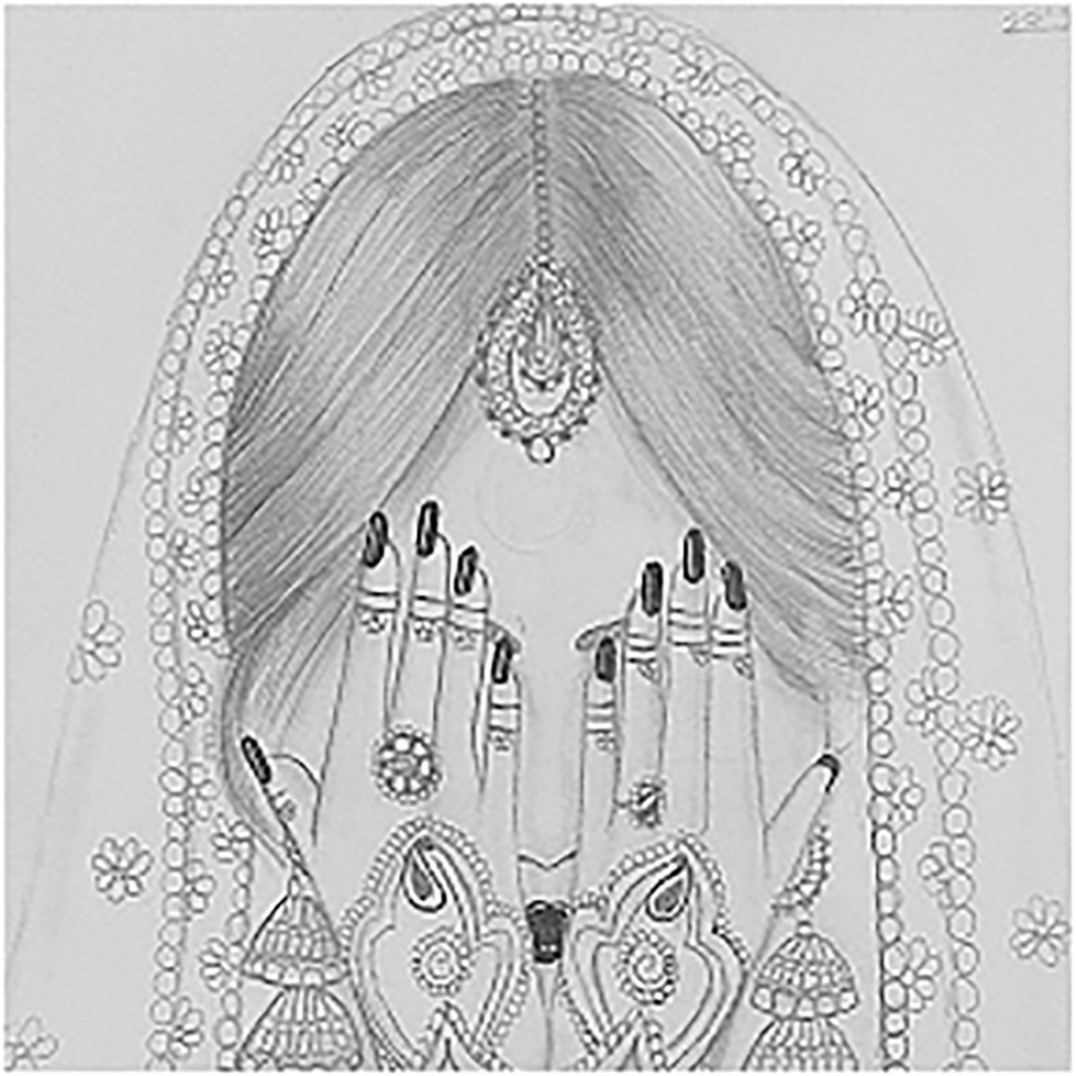
Bride.

**Fig 6:**
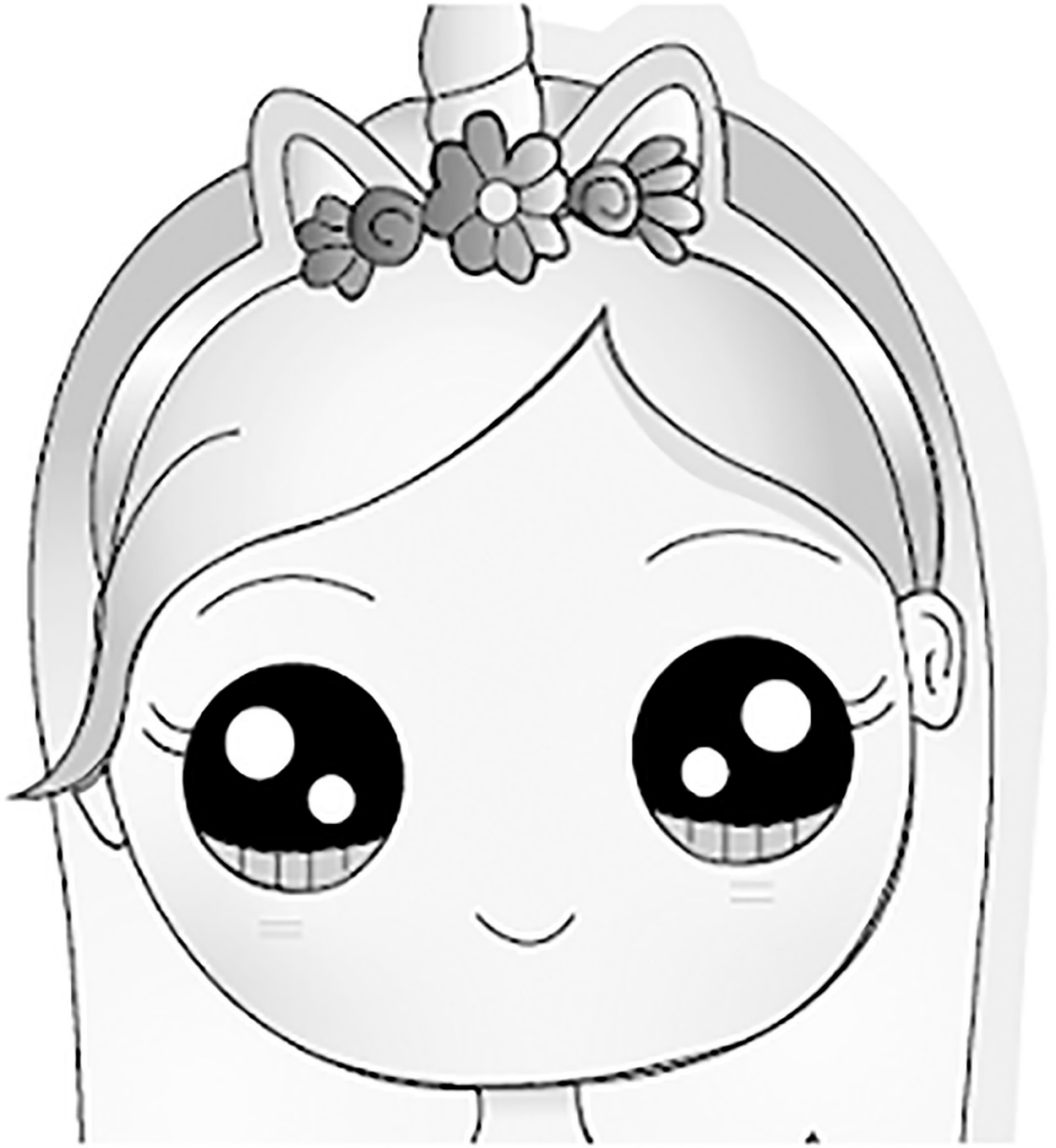
Doll.

**Fig 7:**
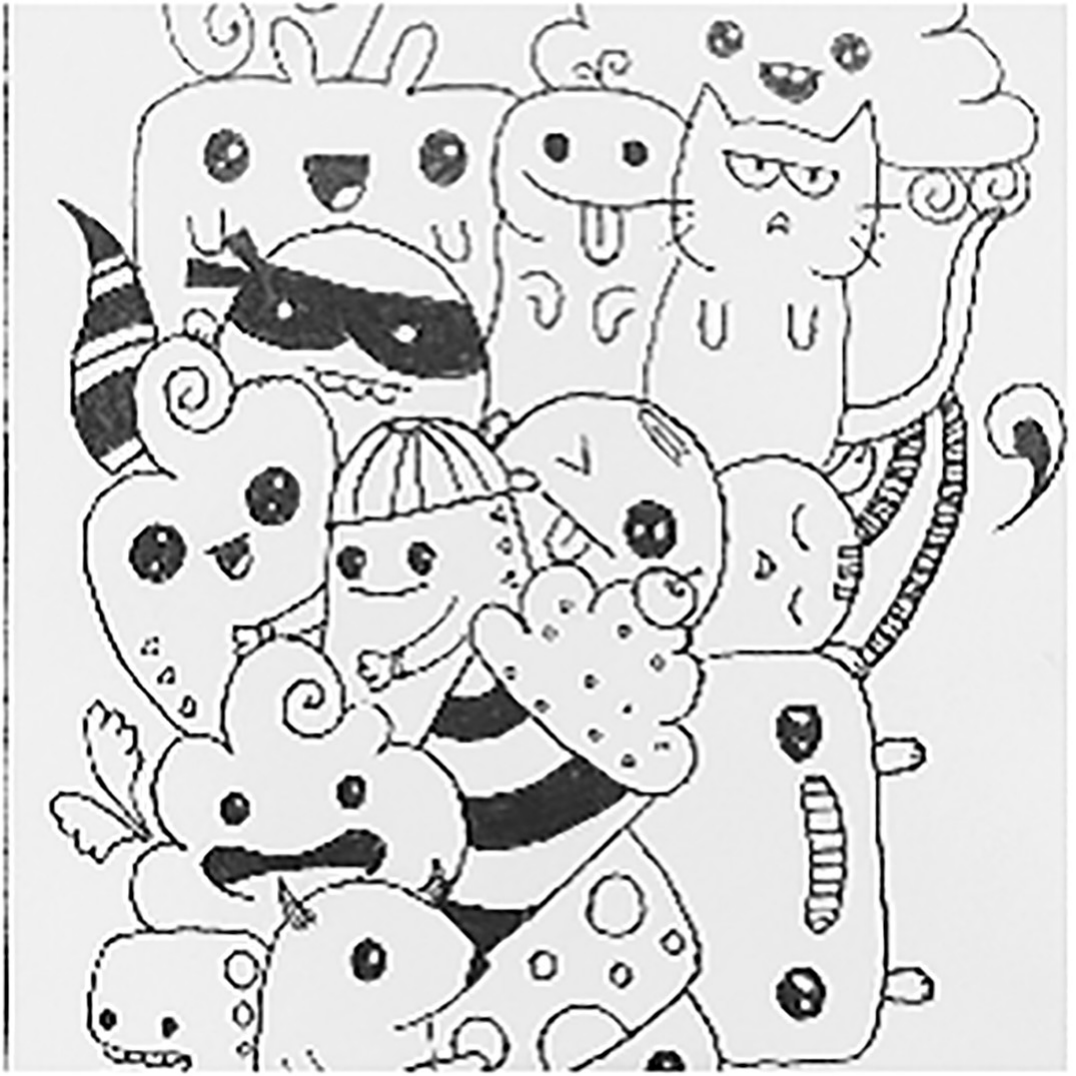
Doodle.

**Fig 8:**
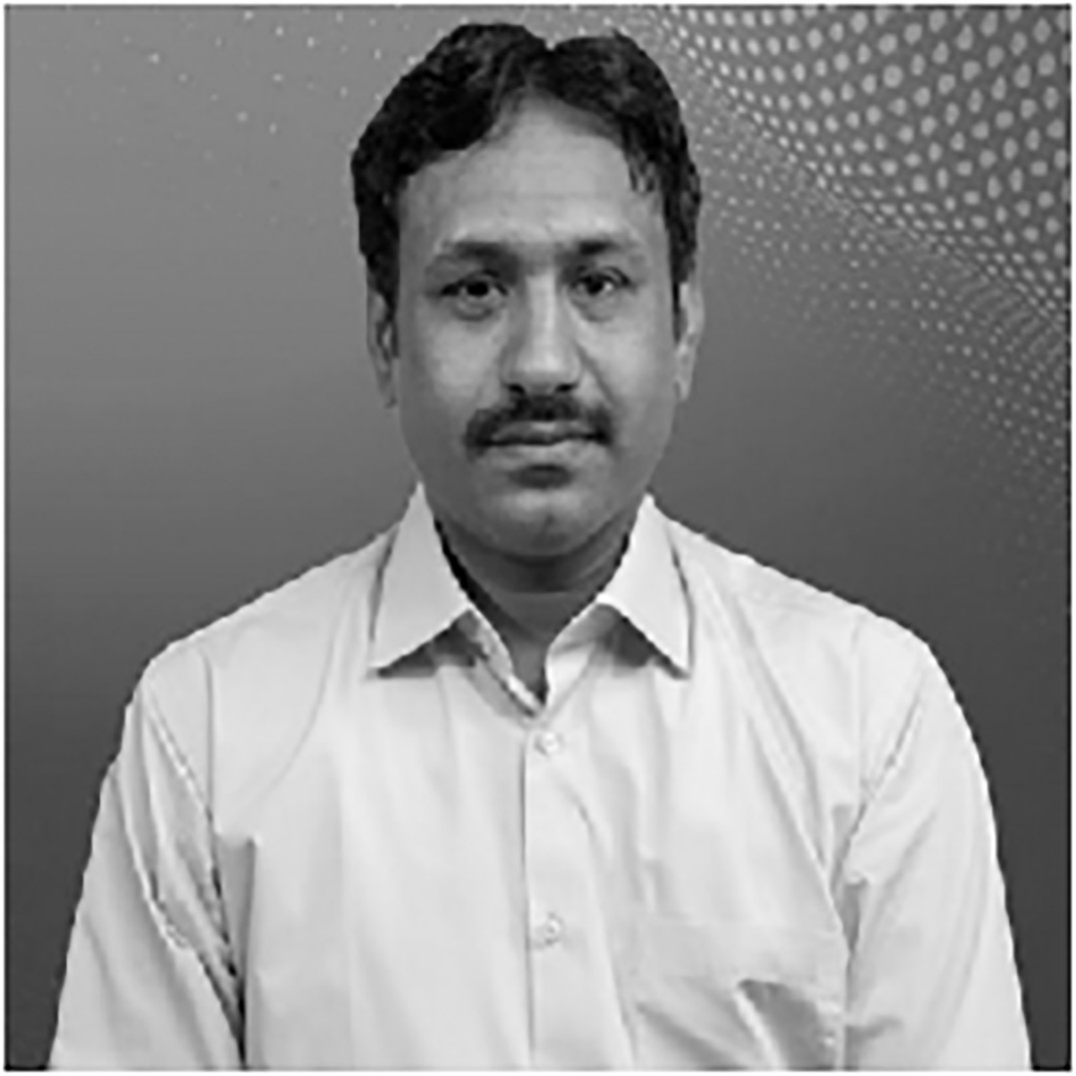
Khizar.

**Fig 9:**
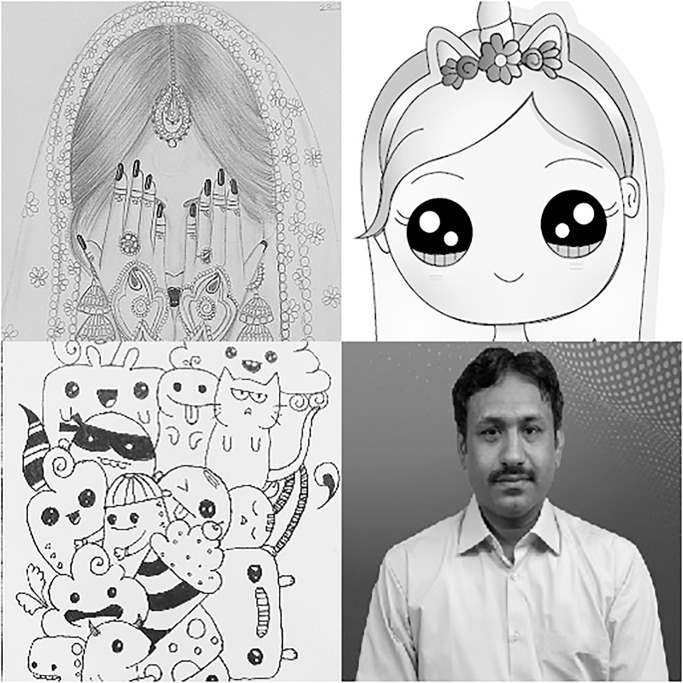
The combined test image.

**Fig 10:**
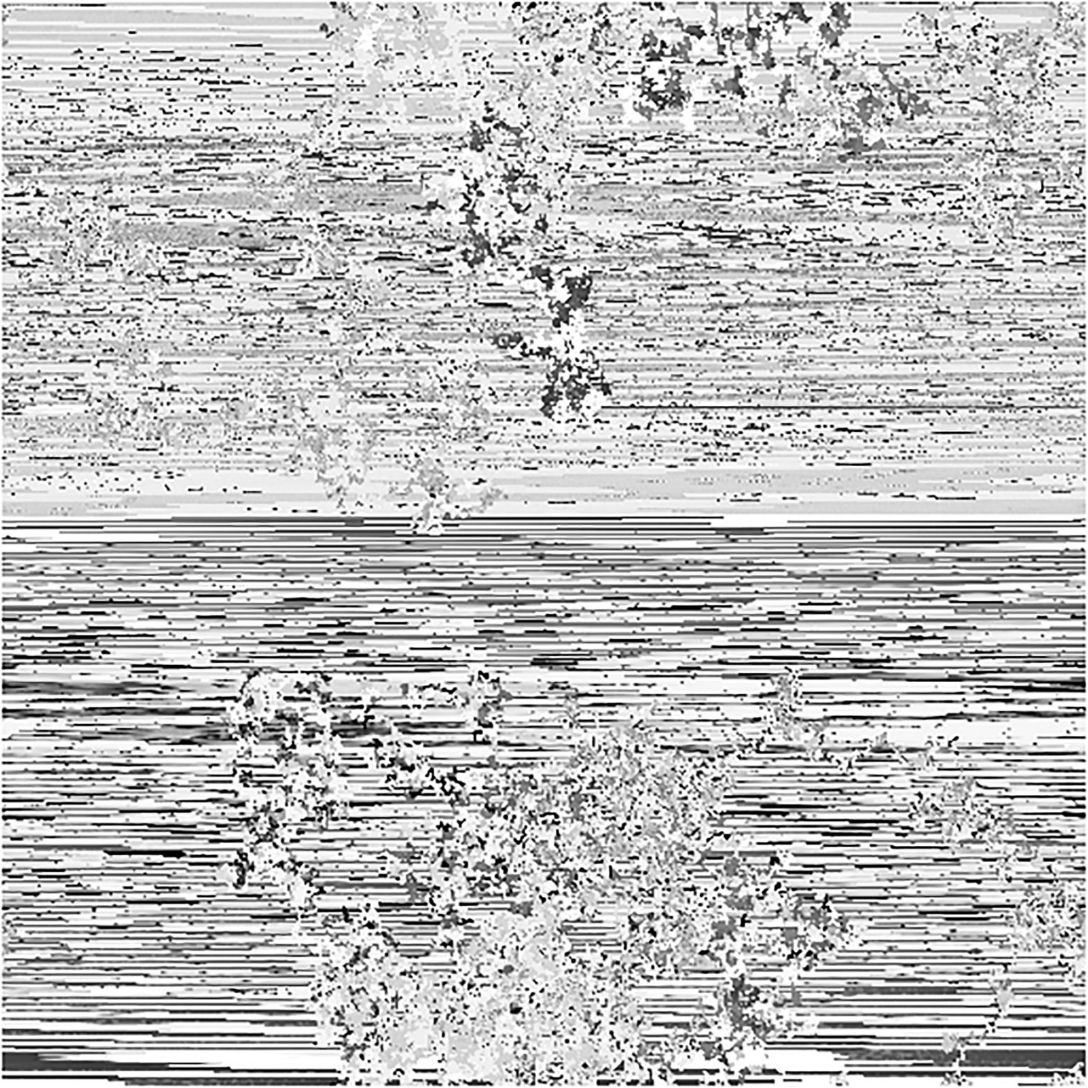
The combined test scrambled image.

**Fig 11:**
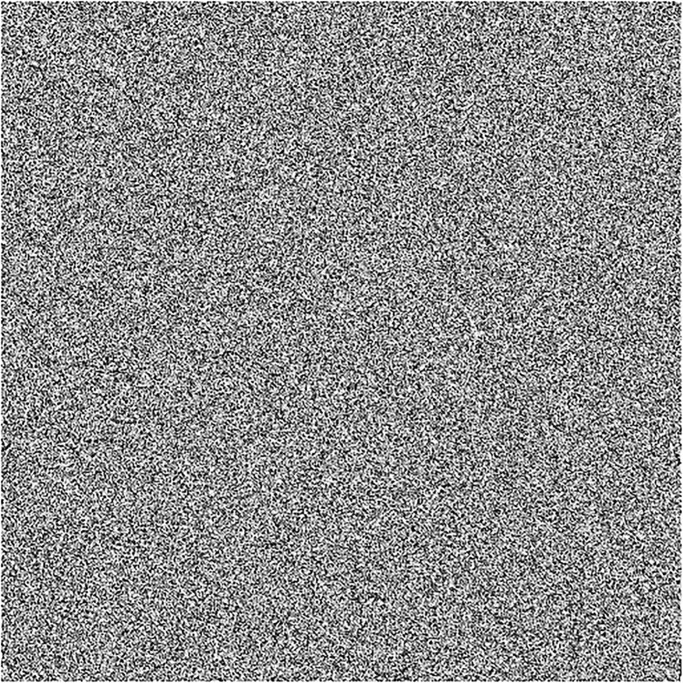
The combined test cipher image.

**Fig 12:**
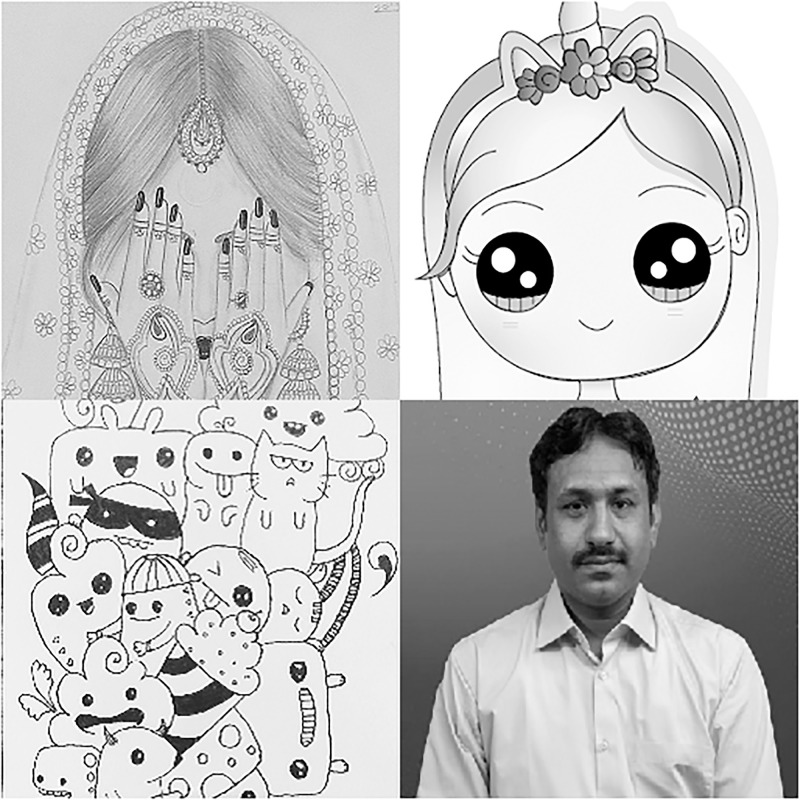
The decrypted test images.

In this section, we will give a demonstration by taking some sample images for the multi-image cipher described in the previous section. The images chosen for this purpose are Lena, Brain, Couple and Butterfly all with the sizes of 256 × 256. These images have been taken from the USC-SIPI Image Database. Along with 64-bit double-precision, MATLAB 2018 version according to the IEEE [19] standard 754 has been employed for the purpose of simulation. The chaotic Henon map has been sparked by giving these values: *x*_0_ = 0.3, *y*_0_ = 0.3, *a* = 1.3, *b* = 0.2. Besides, the initial values and the system parameters for the Blum Blum Shub pseudorandom number generator are *p* = 8616460799, *q* = 9848868889, *r*_0_ = 250990001 and *n* = 65536. Figs 5–8 depict the test images of Bride, Doll, Doodle and Khizar. Additionally, the Fig 9 shows the combined test image. As the proposed multiple images cipher is applied over the combined image, we can see its scrambled and cipher versions in the Figs 10 and 11 respectively. One can easily appreciate the do-ability of the proposed multiple image cipher that the cipher images have been completely converted into the unrecognizable and the cloudy format after the application of the algorithm over them. Besides, the algorithm has also successfully retrieved the original plain images (Fig 12) which signals towards the successful implementation of the decryption machinery of this study as well.

## 5 Validation

Researchers have employed a plethora of validation metrics to authenticate the image ciphers. In this section, suggested image encryption scheme would be thoroughly subjected to these metrics. Every metric sheds light on some facet of the security. Apart from that, these research works [6, 7, 13, 20] have been chosen in order to do a comparative analysis between them and the proposed work. It is to be noted that comparison and ensuing discussion would be carried out in the dedicated Section 6.

### 5.1 Analysis of key space

The size of the key space of any security product has a direct bearing upon the potential brute force threat. Adversaries and other cryptanalytic savvy generate all the possible secret keys in a systematic fashion in this attack dynamics to reach to the real secret key. But, if the key space is large enough that they could not generate it in a practical time then this threat may be easily thwarted. The proposed scheme has used the Henon chaotic map which has four variables whose starting values serve as the secret key. These four variables are *x*_0_, *y*_0_, *a* and *b*. The precision of the system on which the current project has been carried out is 10^−15^. The key space comes out to be (10^15^)^4^ = 10^60^ ≈ 2^199^. This value is 2^199^ >> 2^100^ which is the minimum threshold set by the cryptographers to avert the potential threats of brute force attack [1]. Besides, if the variables *p*, *q*, *r*_0_ and *n* being employed in the BBS are used, then the key space would have been even more than that 2^199^. Hence the proposed cipher is immune to this important attack.

#### 5.1.1 Randomness analysis of the Combined cipher image

The inspection of the cloudy and unrecognizable format of the cipher image given in Fig 11 through mere a naked eye is not sufficient, rather, there must be an objective and impartial yardstick to gauge the randomness and arbitrariness in the pixels of cipher images. Luckily, there exists a NIST Test Suite [21] for this purpose. In order that the randomness of the given bit sequences to be accepted, significance level *p* for the different tests should surpass the threshold value of 0.01. The test results for the Combined cipher image (Fig 11) are shown in the Table 1. We can note that all the values of these results exceed the threshold value of 0.01 which is indicative to the fact that the pixels of the resultant Combined cipher image are sufficiently randomized.

**Table 1:** Statistical randomness test results for the different *p* values of the streams against the cipher image of Fig 11.

Name	*p*−*value*	Result
Frequency	0.358256	✓
Block Frequency (*m* = 128)	0.599710	✓
Cumulative Sums (Forward)	0.217643	✓
Cumulative Sums (Reverse)	0.440087	✓
Runs	0.048729	✓
Longest Run	0.223871	✓
Rank	0.427611	✓
FFT	0.598712	✓
Non Overlapping Template (*m* = 9, *B* = 000000001)	0.029001	✓
Overlapping Template (*m* = 9)	0.269812	✓
Universal Statistical Test	0.039812	✓
Approximate Entropy	0.879812	✓
Random Excursions	0.810965	✓
Random Excursions Variant	0.641287	✓
Serial (*m* = 8)	0.360081	✓
Linear Complexity	0.374412	✓

### 5.2 Statistical analysis

Researchers employ histogram and correlation analyses under this heading.

#### 5.2.1 Histogram

**Fig 13:**
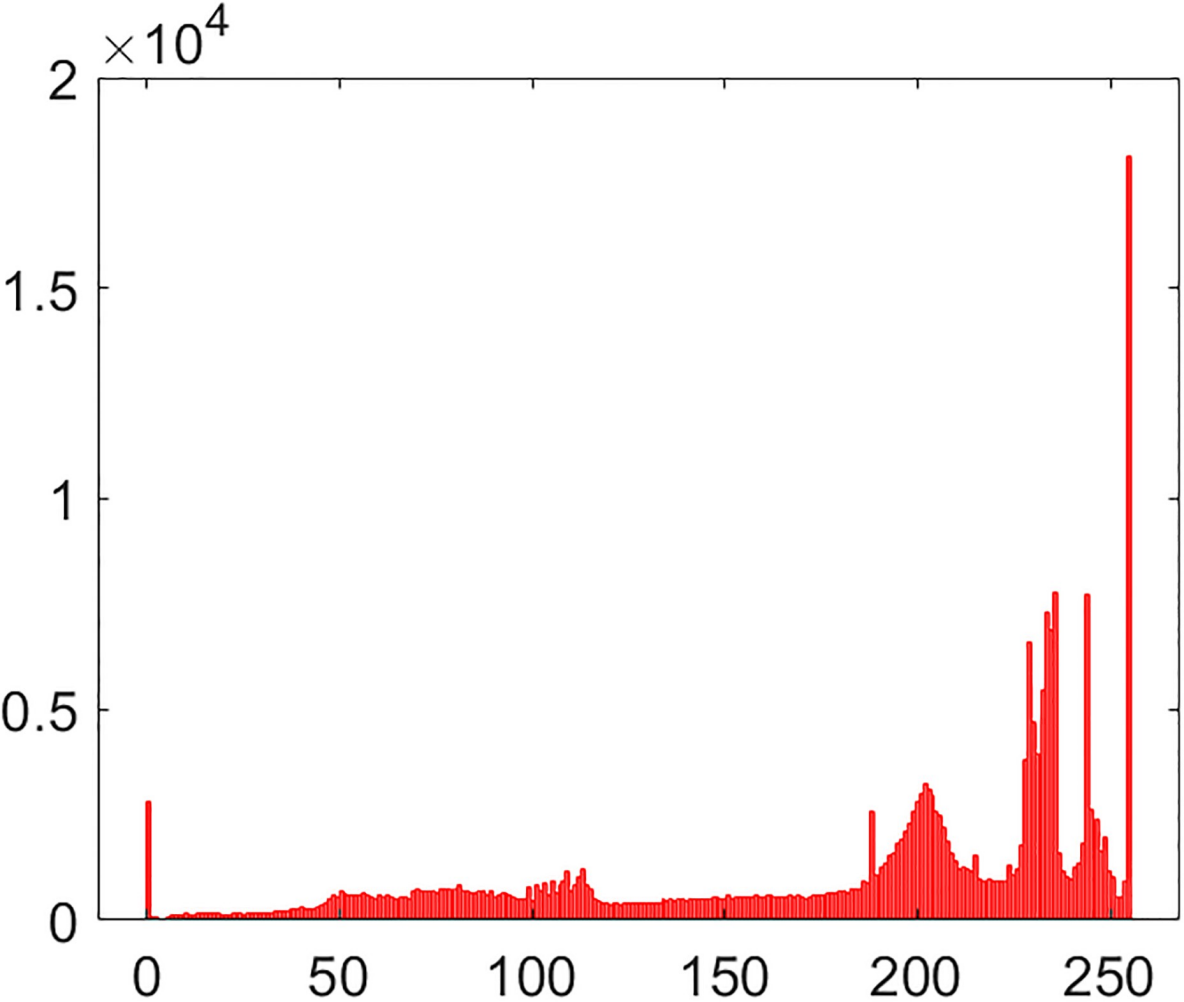
Histogram of combined test plain image.

**Fig 14:**
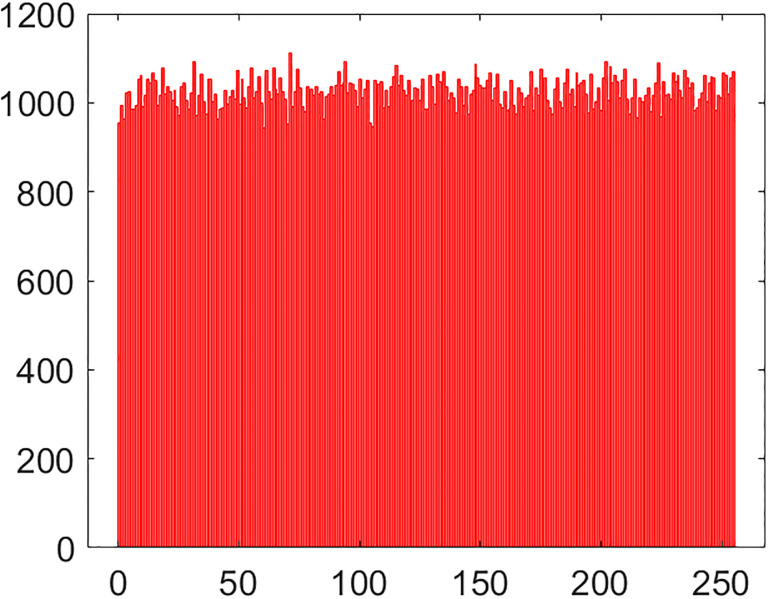
Histogram of combined test cipher image.

Histogram is a frequently used instrument in image processing paradigm. This is a very handy way to look at the number of intensity values for each pixel in the given image. As the histogram of some plain image is drawn, its bar undergoes a sharp twist up and down which is replete with the information about plain image which, in turn, fascinates a lot to the cryptanalytic savvy. After the plain image is encrypted through the application of encryption algorithm over it, its histogram has a very smooth and uniform looking bar over it. The more smooth the bar a histogram has, the more secure it is from the histogram attack from the hackers’ community. Histograms of cipher and plain images of combined test image are drawn in the Figs 13 and 14. As it is obvious, that bar evolves in a slating fashion against the plain image. Whereas, it remains very uniform for the given encrypted image. This bar’s uniformity of the histogram is symptomatic to the fact that the suggested multi-image cipher is furnished with plausible security effects.

#### 5.2.2 Correlation coefficient analysis

This is another frequently used security parameter to judge the performance of an image cipher. Fact of the matter is that the pixels of a normal/plain image are tightly interlinked (correlated) with each other. This interlinking is explored in three orientations/directions of vertical, diagonal and horizontal. As these pixels go through the diffusion and confusion operations of the cipher, this tight correlation drops markedly. This explains why the encrypted image looks like cloudy and noisy. If an image is encrypted to the idealistic proportions, this correlation becomes straightforwardly equal to the nil value. Further, images have thousands of pixels. It is infeasible to take every possible pair from three directions vertical, horizontal and diagonal. So, in this study, 5,000 pairs from the arbitrary locations of both the plain and cipher images have been taken through the random numbers. The following mathematical formula has been employed in this calculation [22]:

$$\begin{array}{c} CC=\frac{N\sum_{j=1}^{N}\left(x_{j}\times y_{j}\right)-\sum_{j=1}^{N}x_{j}\times \sum_{j=1}^{N}y_{j}}{\sqrt{\left(N\sum_{j=1}^{N}x_{j}^{2}-{\left(\sum_{j=1}^{N}x_{j}\right)}^{2})(N\sum_{j=1}^{N}y_{j}^{2}-{\left(\sum_{j=1}^{N}y_{j}\right)}^{2}\right)}} \end{array}$$ (5)

**Fig 15:**
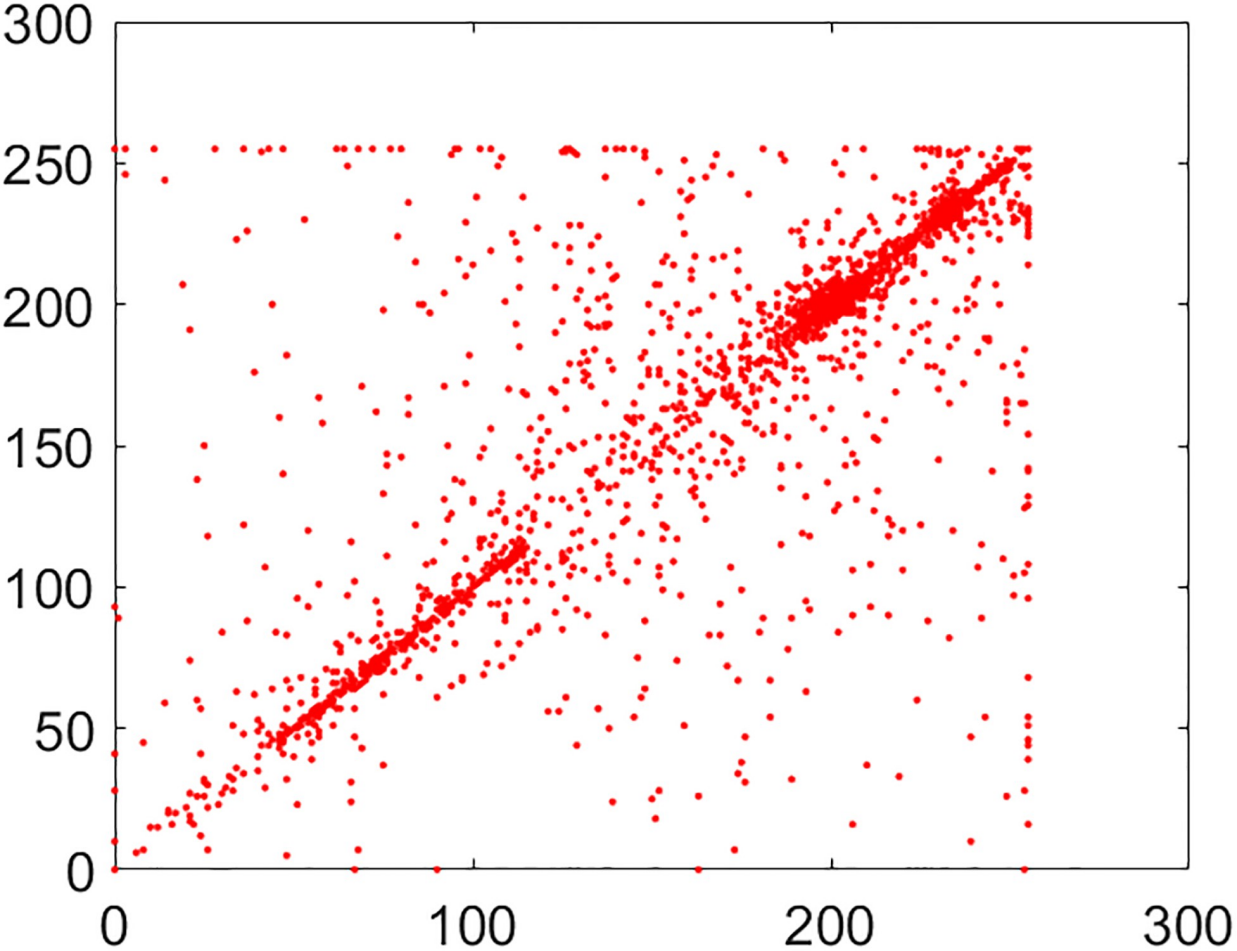
Correlation distribution: Combined test plain image in horizonal direction.

**Fig 16:**
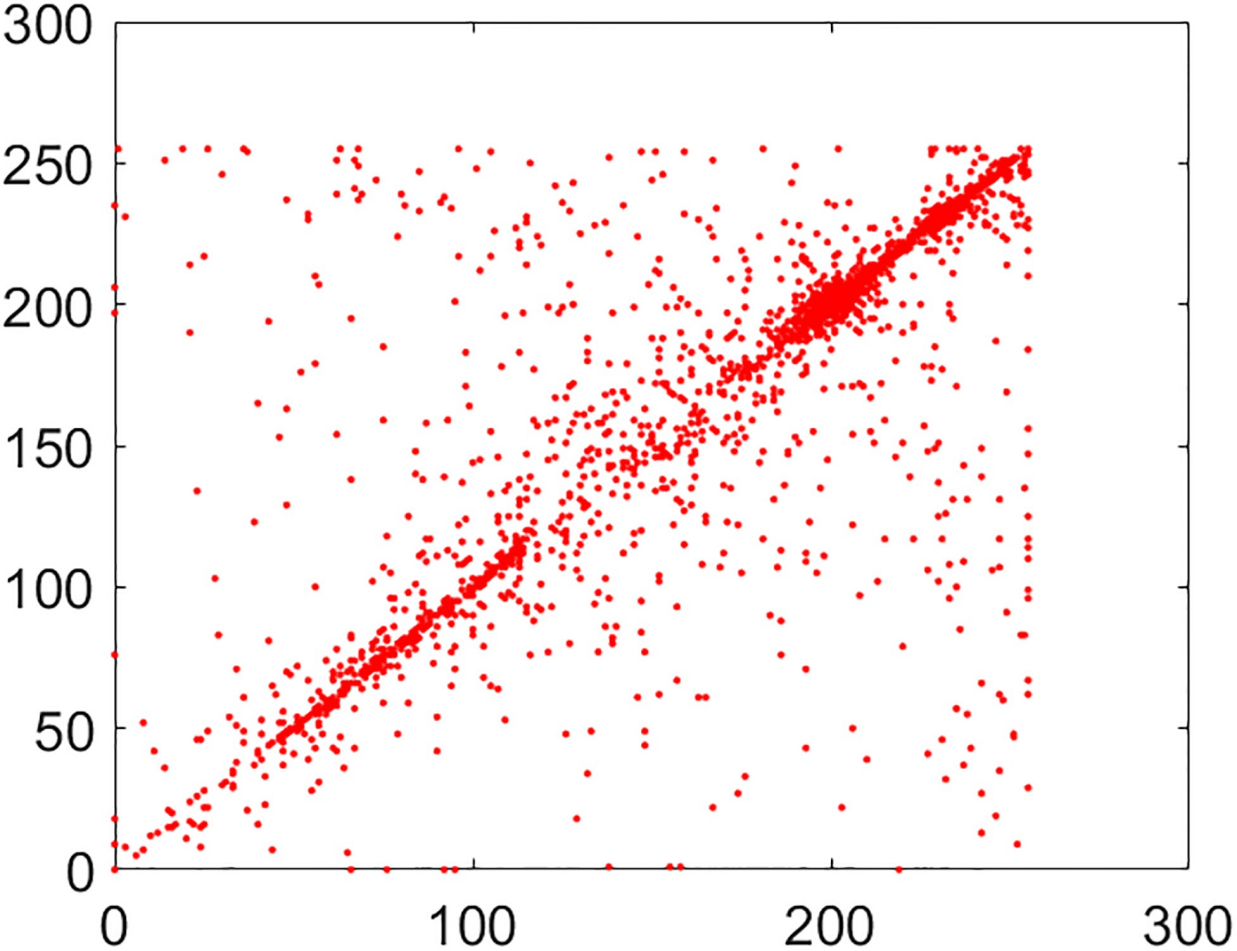
Correlation distribution: Combined test plain image in vertical direction.

**Fig 17:**
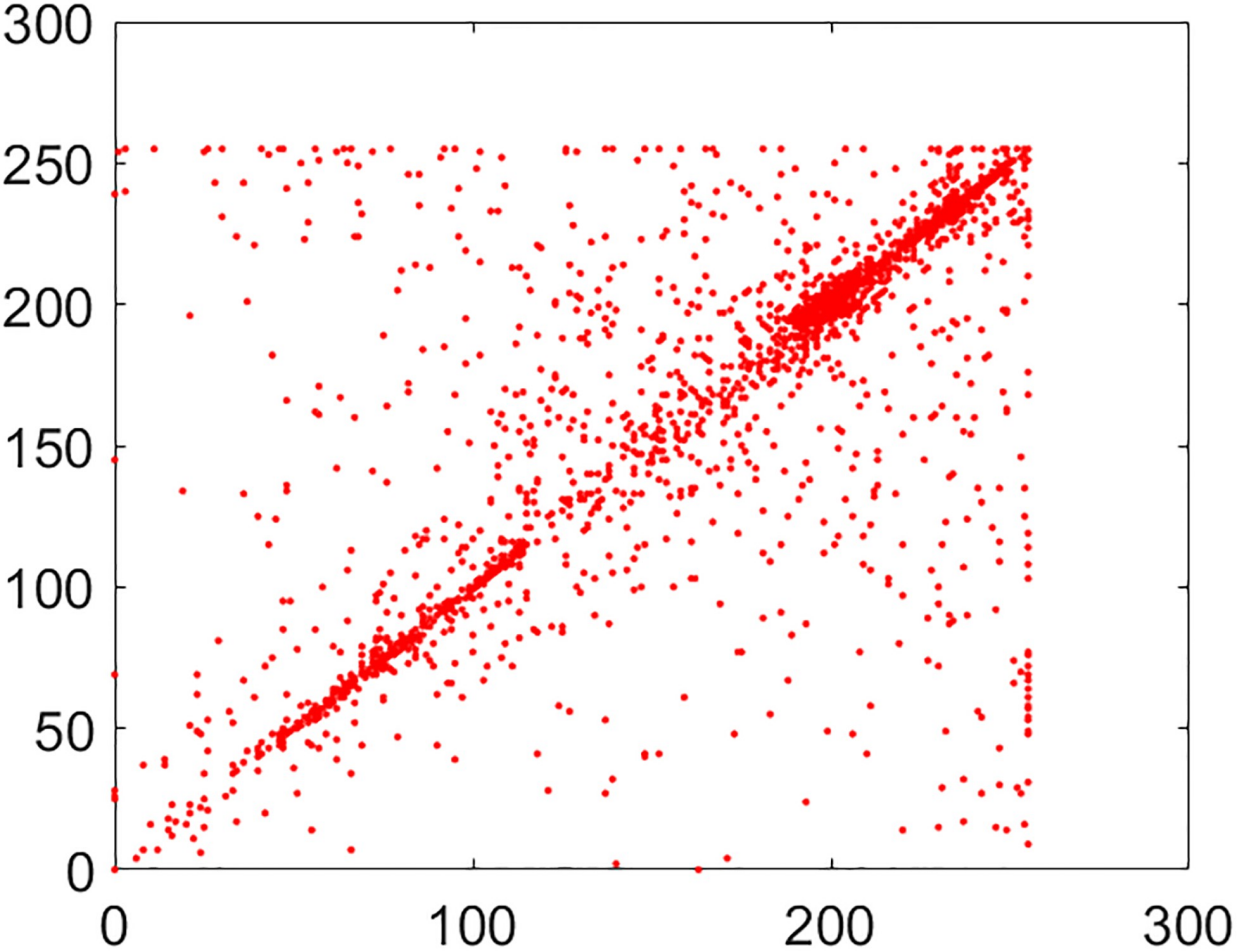
Correlation distribution: Combined test plain image in diagonal direction.

**Fig 18:**
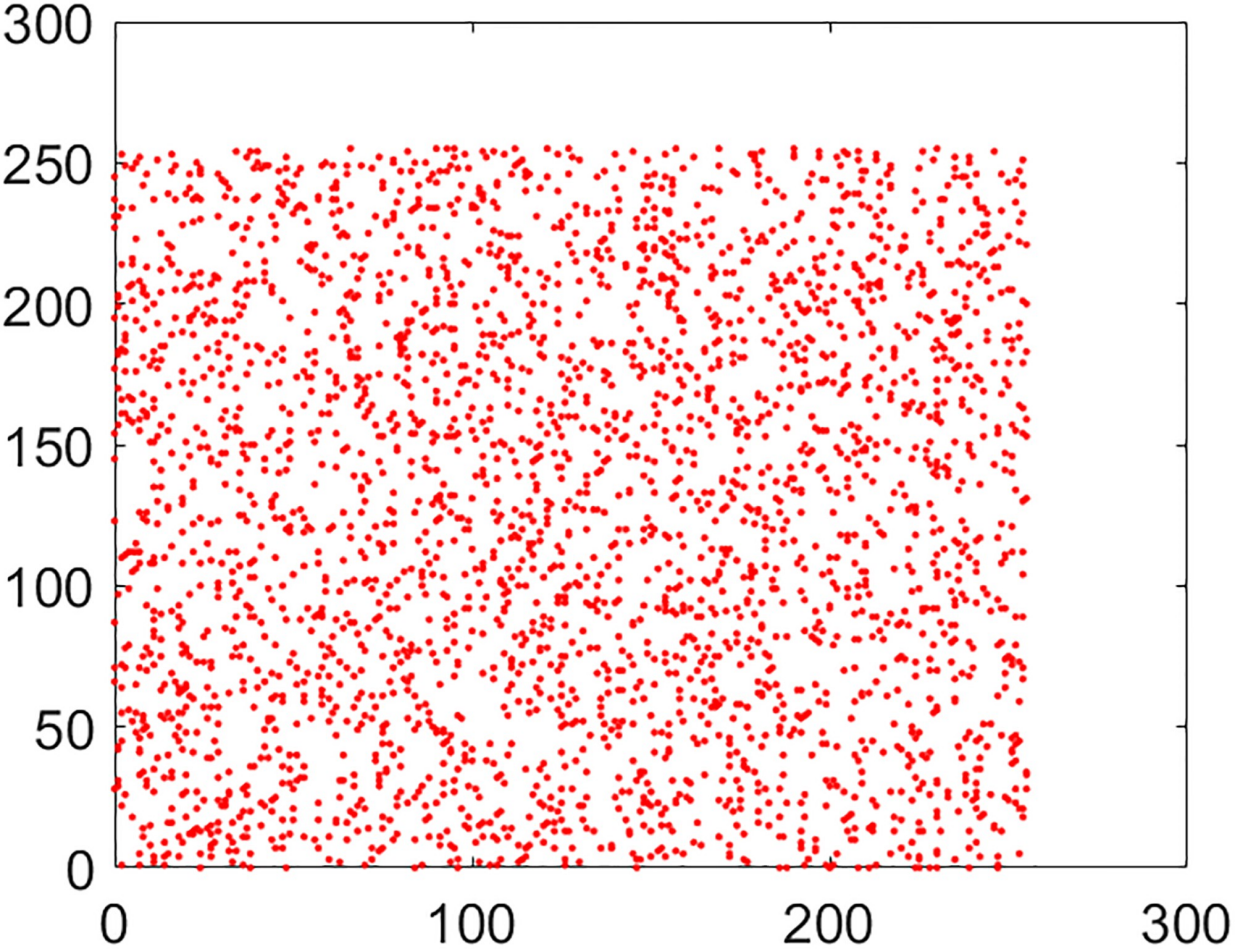
Correlation distribution: Combined test cipher image in horizonal direction.

**Fig 19:**
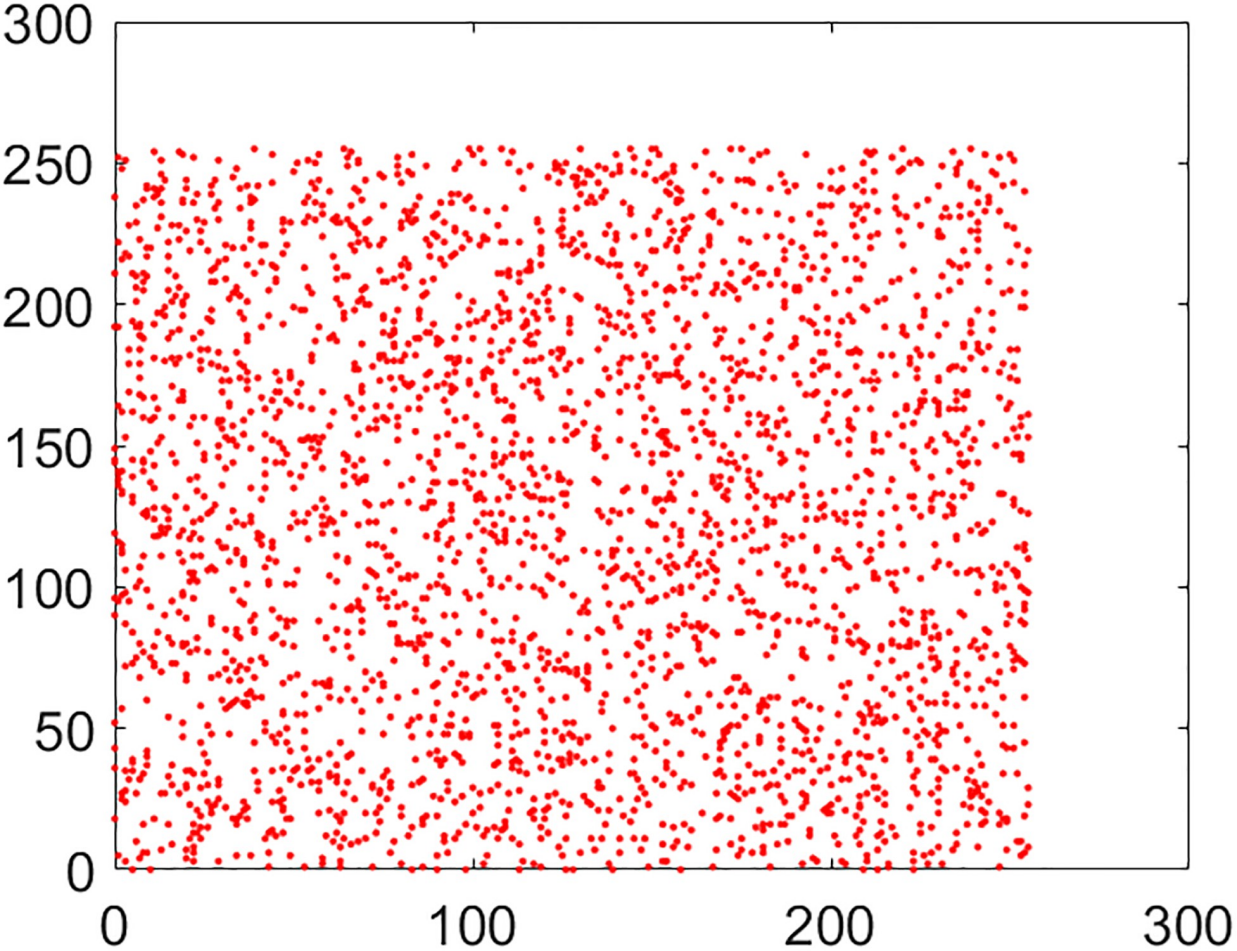
Correlation distribution: Combined test cipher image in vertical direction.

**Fig 20:**
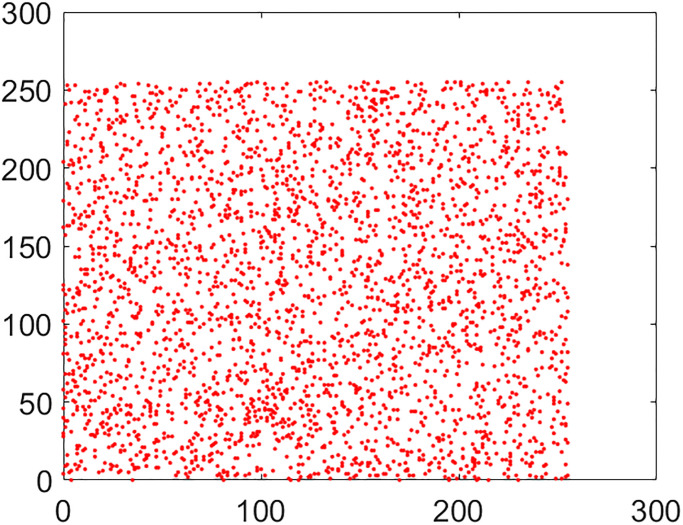
Correlation distribution: Combined test cipher image in diagonal direction.

In this equation, *x* and *y* refer to color codes (intensity values) of two consecutive pixels. Here *N* denotes the number of pixels present in the given image. Correlation distribution of vertically, diagonally and horizontally neighboring pixels of original and encrypted test images are shown in Figs 15–20.

The correlation coefficients between two adjacent pixels for original and encrypted combined test image have been given in Table 2.

**Table 2:** Correlation coefficient between the original and corresponding cipher images.

Image	Correlation direction		
	Horizontal	Vertical	Diagonal
Combined test plain image	0.9209	0.9154	0.9065
Combined test cipher image	0.0087	0.0064	-0.0041

From the Table 2, we can see that the correlation coefficient for the combined plain image is nearly equal to 1 while this metric drops down nearly equal to zero in case we have encrypted image.

### 5.3 Analysis of information entropy

This validation metric assumes a great importance while development of any security product. Reason for this is that randomness and arbitrariness lie at the very heart of the enterprise of cryptography. This is such an instrument through which we can measure the degree of randomness some data/image may have. As the cipher is applied over some plain image, its pixels undergo a sea change in their relative positions as well as in their intensity values. The extent with which these pixels get disturbed is measured by the cryptographers through this yardstick. It was Shannon [23] who developed a mathematical formula to measure this concept in 1949:

$$\begin{array}{c} Z\left(r\right)=\sum_{c=0}^{2^{n}-1}p\left(r_{c}\right)log\frac{1}{p(r_{c})} \end{array}$$ (6)

In the above equation, *Z*(*r*) denotes the information entropy of some image *r*. Besides, *p*(*r*_*c*_) refers to the probability of symbol *r*_*c*_. The maximum value of this metric comes out to be 8 for some image with 256 gray levels. This can be happened only when some plain image is both confused and diffused to the idealistic proportions which is perhaps not possible. The more near to 8 the value of this metric comes out, the better it is for the security effects. Table 3 depicts the values of this important metric for both the plain and cipher images. We can see that its value is nearly equal to 8 in case of cipher image which is an indication of nice security effects. So, we are justified in saying that the proposed image cipher is immune to the attack of entropy.

**Table 3:** The results of information entropy analysis.

Encryption algorithm	Image	Original	Encrypted
Proposed	Combined test image	7.7612	7.9993

### 5.4 Differential attack

Cryptanalysts have a large array of attacks to break ciphers. This attack falls on one of them. In this attack dynamics, two sample test images are taken by hackers. One is the straightforward image and another has some very minute change, say the change of intensity value in just one pixel of plain image. Now both of these images are encrypted. A microscopic observation of these two images has the potential to lead the hackers to spot some meaningful relationship between these two images. A further struggle may cause to the revelation of the secret key. This threat is normally tackled through the two security parameters of *NPCR* and *UACI*. The former corresponds to the number of pixels change rate and the latter to the unified average changing intensity. The mathematical formulae of these concepts are

$$\begin{array}{c} NPCR=\frac{\sum_{l,m}D\left(l,m\right)}{F\times G}\times 100\% \end{array}$$ (7)

where *F* and *G* represent the width and height of the image respectively. *D*(*l*, *m*) can be defined by:

$$\begin{array}{c} D\left(l,m\right)=\{\begin{array}{cc} 1, & \text{if}\;\;\;C\left(l,m\right)\neq C^{\prime }\left(l,m\right);\\ 0, & \text{if}\;\;\;C\left(l,m\right)=C^{\prime }\left(l,m\right). \end{array} \end{array}$$ (8)

$$\begin{array}{c} UACI=\frac{1}{F\times G}[\sum_{l,m}\frac{|C\left(l,m\right)-C^{\prime }\left(l,m\right)|}{255}]\times 100\% \end{array}$$ (9)

*C* and *C*′ are respectively the ciphered images before and after one pixel of the plain image is changed. The values for the security parameters of the differential attack, *i.e*., *NPCR* and *UACI* have been drawn in the Table 4.

**Table 4:** Results of NPCR and UACI for the combined test image.

Algorithm	NPCR(%)	UACI(%)
Proposed	99.6312	33.4508

One can see that these values are very close to the ideal values of these two metrics which once again confirms the prowess and robustness of the proposed image cipher against the potential differential threats.

### 5.5 Peak-signal-to-noise-ratio analysis

This is the fundamental principle of the niche of image encryption that a maximum discrepancy may be created between the plain image and its cipher version through the two principal operations of confusion and diffusion. This discrepancy/difference is objectively measured through a yet another security parameter called peak-signal-to-noise-ratio (*PSNR*). Its mathematical equation is

$$\begin{array}{c} \left\{ \begin{array}{c} PSNR=20log_{10}\left(255\sqrt{MSE}\right)dB\\ MSE=\frac{1}{F\times G}\sum_{l=1}^{F}\sum_{m=1}^{G}{\left(Q_{0}\left(l,m\right)-Q_{1}\left(l,m\right)\right)}^{2} \end{array}\right. \end{array}$$ (10)

where *F* and *G* refer to the size of the test image. *Q*_0_(*l*, *m*) and *Q*_1_(*l*, *m*) are the intensity values of the pixels for the original and encrypted images. Besides, *MSE* refers to the mean squared error which exists between the original image and the encrypted image. Larger value of *MSE* corresponds to the better security. Since *PSNR* and *MSE* are reciprocally interrelated, so larger value of *MSE* would cause to produce relatively smaller value of *PSNR*. Hence relatively smaller value of *PSNR* indicates the good security effects. Table 5 shows the *PSNR* values given by the published works. This security parameter’s value is infinite (∞). We can infer that no change exists in the original image and the decrypted image. We can also conclude that the proposed image cryptosystem is lossless in nature. Besides, the *PSNR* value obtained by our algorithm is better than those in [24–26].

**Table 5:** The *PSNR* results of the original images/decrypted images: Two lettered abbreviation ‘O-C’ denotes the original-ciphered images, and ‘O-D’ the original-decrypted images.

		Combined test image
Ours	(O-D)	∞
	(O-C)	8.2173
Ref. [24]	(O-D)	96.2956
	(O-C)	9.0348
Ref. [25]	(O-C)	8.6878
Ref. [26]	(O-C)	9.0486
Ref. [27]	(O-C)	8.1300
Ref. [28]	(O-C)	8.5581

### 5.6 Noise and data loss/crop attacks

**Fig 21:**
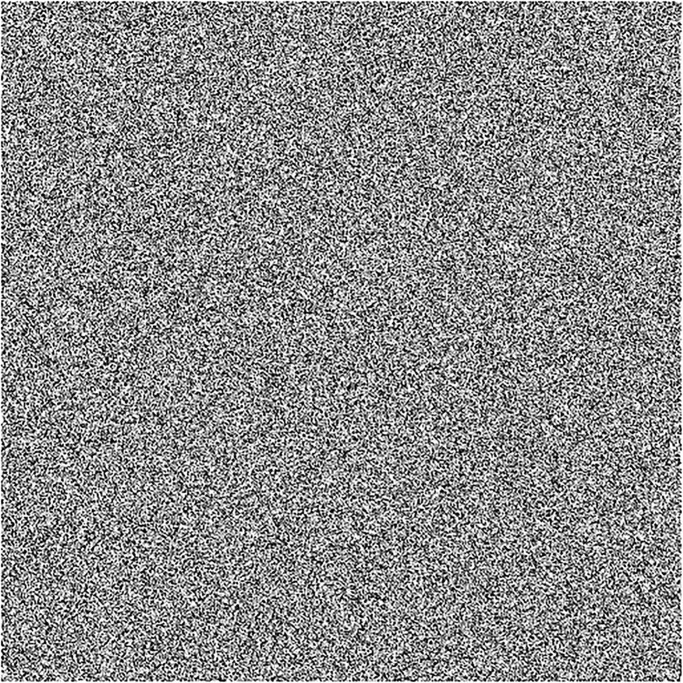
Pepper & Salt noise attack on the encrypted image with the noise density 0.1.

**Fig 22:**
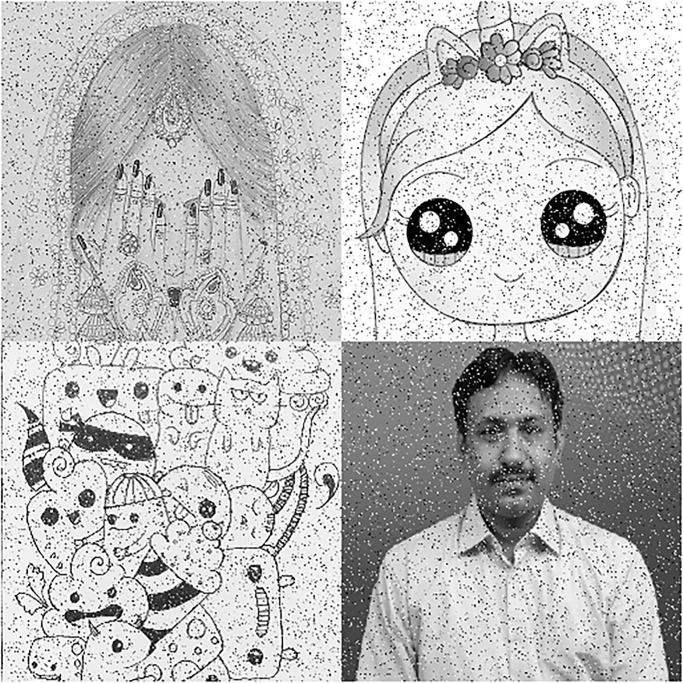
Decrypted image from Fig 21.

Accidents and other uncertain events characterize the general tone and tenor of the real world. This is what happens with the cipher images as they are stored on some electro-mechanical gadget or are transferred through some public network. In particular, they come under the threats of noise attack and data crop attack. For demonstrating the defiance of the proposed work against these two potential threats, we have artificially added Pepper & Salt noise. The amount of added noise is 0.1 to the cipher image drawn in the Fig 21. Upon subjecting it to the decryption algorithm, the obtained image has been drawn in the Fig 22. The decrypted image can be easily recognized. This points to the fact that the suggested image encryption scheme has the potential of thwarting the noise attack.

**Fig 23:**
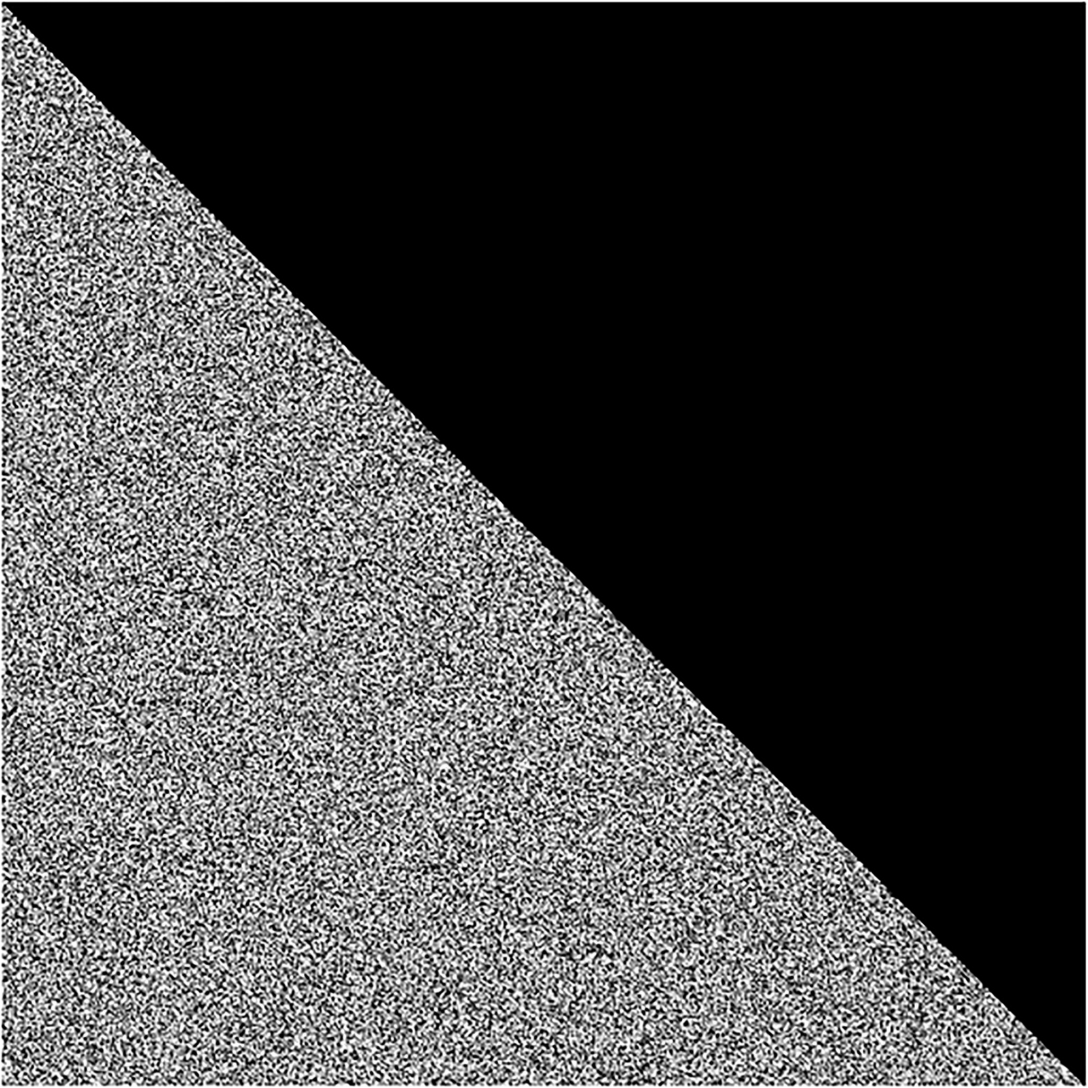
Data loss attack: Encrypted test image with an attack of the triangle with area 12×512×512.

**Fig 24:**
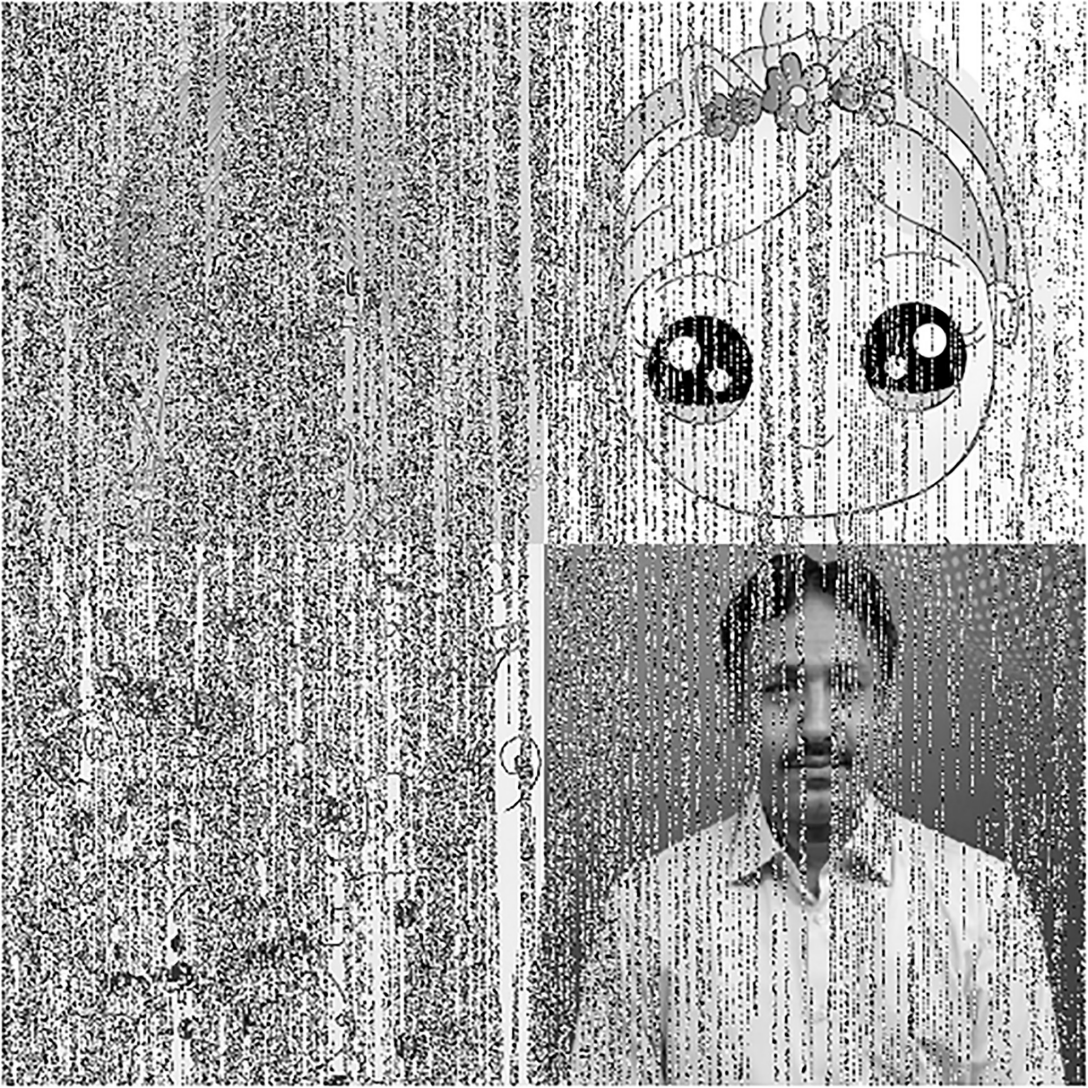
Decrypted image from Fig 23.

Besides, in order to show the capability of the proposed image cipher of withstanding the data crop attack, the cipher image has been drawn in the Fig 23 with attack of data loss. Later on, in order to recover the original plain image, this cropped cipher image has been decrypted by applying the decryption algorithm over it. Fig 24 plots the restored image. This plotted image asserts that the potential image cryptosystem has the ability to endure the data crop attacks as well.

### 5.7 Speed performance and complexity analysis

The suggested image cryptosystem has been written under 64-bit Operating System, x64-based processor, RAM = 8.00 GB, Intel(R) Core(TM) i7–3740QM CPU @ 2.70GHz. In academia and industry, the problem of assessing the computational speed of some algorithm has been approached in two ways. We will discuss both of these approaches one by one here. In the first approach, the given algorithm is submitted to the machine and its response time is noted through some gadget like stopwatch. The Table 6 shows that the combined image got converted to its cipher version in just 0.2276 seconds.

**Table 6:** Encryption speed of the proposed algorithm.

Algorithm	Image	Speed (sec)	Mbit/sec
Proposed	Combined test image	0.2278	5.5782

Besides the speed of the encryption algorithm, normally another metric is also employed to characterize the time spent and the amount of image to be encrypted. This idea has been thrown through the notion of encryption throughput (*ET*). It is a sort of “rate” of the cipher. Below is its mathematical formula.

$$\begin{array}{c} ET=\frac{Image_{size}\left(Bits\right)}{Encryptione_{time}\left(seconds\right)} \end{array}$$ (11)

The last column of Table 6 shows that the encryption throughput for the proposed cipher is 5.5789 Mbit/seconds which is again very promising.

The inherent drawbacks and other problems which plague this approach are worth mentioning. Firstly, the computational time varies with each input plain image. Secondly, this time depends heavily upon the underlying hardware. Thirdly, it also depends upon the operating system and the compiler through which it is running. The second approach called the theoretical approach addresses all these problems. This approach carries us to such a setting where we get the pure intrinsic speed of the algorithm. Asymptotics [29] —a theory of mathematics is normally employed in this approach in order to analyze the given algorithms.

The time complexity of Algorithm 1 is *O*(*n*) where *n* is the number of iterations for the BBS random number generator. Moreover, the complexity of Algorithm 2 is *O*(2*MN*) where (*M*, *N*) are the dimensions of the combined test image. The analysis of Algorithm 3 is rather more elaborative. The complexity of lines (3–5) is *O*(*MN*). The complexity of lines (6–7) is *O*(*N*). Moreover, lines (8–16) contribute *O*(8*MN*) to the time complexity. Lastly, the complexity of lines (18–24) is *O*(5*MN*). By adding all these complexities and ignoring the lower order terms, we get the time complexity of the proposed algorithm to be *O*(16*MN*) which is better than *O*(24*MN*) [27, 30] and *O*(64*MN*) [31].

## 6 Comparison and discussion

Table 7 provides a panoramic view about the results of the proposed work and those of some other selected researches. Key space of suggested work is 10^60^ which crosses minimum threshold of 2^100^ set by the cryptographers in order to withstand the potential brute force attack. Unluckily, we couldn’t beat a single work based on this metric. Besides, the correlation coefficient results of the proposed work are very nearly equal to zero. The comparison is not straightforward. These results are comparable to the other selected works [6, 7, 13, 20], however. Moreover, the information entropy of the suggested work is 7.9993 which is approximately equal to the ideal value of 8. Our value is better than the one given in the work [13]. Actually, again the comparison is not so smooth and straightforward since the values of entropy naturally get bigger for the larger images. As far as the differential attack is concerned, the proposed work beats all the selected researches [6, 7, 13, 20] based on the metric of NPCR. However, we could only beat [13] based on the metric of UACI. Additionally, the speed of the proposed multi-image cipher is 0.2278 seconds which is better than all the selected works. Further, the encryption throughput is also very nice, i.e., 5.5782 MBit/Sec. Apart from that, the proposed cipher is defiant to the potential noise and data crop attacks as has been demonstrated in the previous section.

**Table 7:** A comparison of the proposed scheme with other schemes.

Schemes	Size and number of images	Key space	Correlation coefficient			Entropy	NPCR	UACI	Speed (Sec)	ET MBit/Sec
			H	V	D					
Ref. [20]	1280 × 1280(16 × 320 × 320)	3.94 × 10^213^	0.0013	-0.0035	-0.0046	7.9999	0.996037	0.334597	5.0565	-
	1024 × 1024(16 × 256 × 256)	-	0.0047	0.0007	0.0051	7.9998	0.996082	0.334570	3.2615	-
	768 × 768(16 × 192 × 192)	-	0.0012	0.0044	-0.0017	7.9997	0.996013	0.334054	1.95	-
	512 × 512(16 × 128 × 128)	-	0.0021	-0.0013	-0.0033	7.9994	0.996081	0.334415	0.9347	-
Ref. [13]	256 × 256 × 4(256 × 256 × 12)	10^105^ ≈ 2^348^	0.0016	-0.0056	0.0015	7.9969	0.996026	0.333636	19.922	-
Ref. [6]	512 × 512 × 4	10^141^	-3.4549 × 10^−7^	1.6199 × 10^−6^	-1.6843 × 10^−7^	7.9998	0.9960655	0.334603	19.1744	-
Ref. [7]	256 × 256 × 4(256 × 256 × 12)	1.1579 × 10^189^	0.0018	-0.0042	0.0041	7.9998	0.996053	0.3348823	0.5299	-
Proposed	256 × 256 × 4	10^60^ ≈ 2^199^	0.0087	0.0064	-0.0041	7.9993	0.996312	0.334508	0.2278	5.5782

Tens of hundreds of ciphers for the single images exist in the literature. Very few researchers worked on the multi-image ciphers. The majority of these ciphers are very time consuming which do not cater to the urgent demands of the current era. By keeping in view this research gap, current work has ventured to provide a new and a speedy multi-image cipher based on the fleet of pawns on the hypothetical chessboard. As has already been described, we got a speed of 0.2278 seconds which is very competitive. Besides, the encryption throughput is 5.5782 MBit/sec. Moreover, the computational complexity of the proposed cipher is *O*(16*MN*) which is better than [27, 30, 31]. Additionally, the proposed work is immune to the frequently launched attacks. These features are vehemently in line with the needs of the current era. Hence, we contend that this new cipher bears rich prospects for some real world application.

## 7 Conclusion

Security of images has become a hot research area in these times. For this purpose, cyber security researchers have paid an increasing attention towards this area. One can find myriads of image ciphers for the security and integrity of single images. These image ciphers deliver very poorly in case some scenario requires the encryption of multiple images in a very short time. This is the situation where multi-image ciphers assume a very important status. Very few multi-image ciphers exist when one surveys the literature. This study has ventured to provide a yet another multi-image cipher to address this need. Our work is based upon the walk of fleet of pawns on a large hypothetical chessboard. As the pawns move in their turn, the pixels from the plain image are transferred to the scrambled image. 2D Henon map has been employed for spawning the random numbers. Plaintext sensitivity has been realized through the SHA-384 hash codes obtained by the given plain images. Besides, the security has been cemented by introducing the Blum Blum Shub random number generator. Comprehensive security and performance analyses indicate that the proposed multi-image cipher has the requisite capacity of thwarting the potential threats from the hackers’ community and expects rosy prospects for some application in industry. In particular, we obtained an information entropy 7.9993 and the encryption throughput 5.5782 MBit/seconds.

## Supporting information

S1 Fig Bride. (PNG)

S2 Fig Doll. (PNG)

S3 Fig Doodle. (PNG)

S4 Fig Khizar. (PNG)
