# Supplementary figures and images for: FOPBIE: Multi-image cipher based on the random walk of fleet of pawns on the large hypothetical chessboard and chaotic system
Source: PLoS One. 2024 Jun 13;19(6):e0295060. doi: 10.1371/journal.pone.0295060 (PMC11175479; doi:10.1371/journal.pone.0295060)

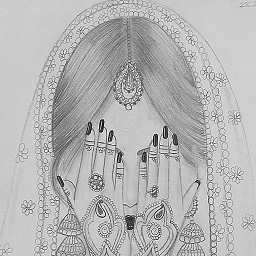

Supplement: S1 Fig — (PNG) [file pone.0295060.s001.png]

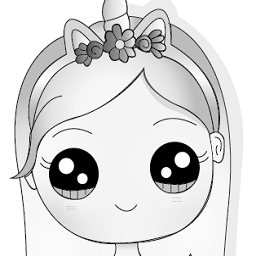

Supplement: S2 Fig — (PNG) [file pone.0295060.s002.png]

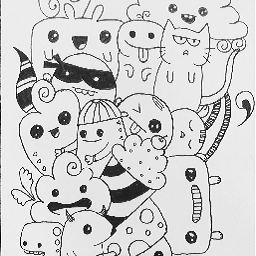

Supplement: S3 Fig — (PNG) [file pone.0295060.s003.png]

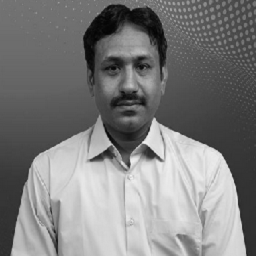

Supplement: S4 Fig — (PNG) [file pone.0295060.s004.png]
